# First synthesis of RuSn solid-solution alloy nanoparticles and their enhanced hydrogen evolution reaction activity†

**DOI:** 10.1039/d3sc06786f

**Published:** 2024-04-16

**Authors:** Xin Zhou, Megumi Mukoyoshi, Kohei Kusada, Tomokazu Yamamoto, Takaaki Toriyama, Yasukazu Murakami, Shogo Kawaguchi, Yoshiki Kubota, Okkyun Seo, Osami Sakata, Toshiaki Ina, Hiroshi Kitagawa

**Affiliations:** a Division of Chemistry, Graduate School of Science, Kyoto University Kitashirakawa-Oiwakecho, Sakyo-ku Kyoto 606-8502 Japan mukoyoshi@ssc.kuchem.kyoto-u.ac.jp kitagawa@kuchem.kyoto-u.ac.jp; b The HAKUBI Center for Advanced Research, Kyoto University Kitashirakawa-Oiwakecho, Sakyo-ku Kyoto 606-8502 Japan; c JST-PRESTO Honcho 4-1-8, Kawaguchi Saitama 332-0012 Japan; d The Ultramicroscopy Research Center, Kyushu University 744 Motooka, Nishi-ku Fukuoka 819-0395 Japan; e Department of Applied Quantum Physics and Nuclear Engineering, Kyushu University 744 Motooka, Nishi-ku Fukuoka 819-0395 Japan; f Center for Synchrotron Radiation Research, Japan Synchrotron Radiation Research Institute (JASRI) SPring-8 1-1-1 Kouto, Sayo-cho, Sayo-gun Hyogo 679-5198 Japan; g Department of Physics, Graduate School of Science, Osaka Metropolitan University Sakai Osaka 599-8531 Japan; h Research Network and Facility Services Division, National Institute for Materials Science (NIMS) 1-1-1 Kouto, Sayo-cho, Sayo-gun Hyogo 679-5148 Japan

## Abstract

Solid-solution alloys based on platinum group metals and p-block metals have attracted much attention due to their promising potential as materials with a continuously fine-tunable electronic structure. Here, we report on the first synthesis of novel solid-solution RuSn alloy nanoparticles (NPs) by electrochemical cyclic voltammetry sweeping of RuSn@SnO_*x*_ NPs. High-angle annular dark-field scanning transmission electron microscopy and energy-dispersive X-ray spectroscopy maps confirmed the random and homogeneous distribution of Ru and Sn elements in the alloy NPs. Compared with monometallic Ru NPs, the RuSn alloy NPs showed improved hydrogen evolution reaction (HER) performance. The overpotentials of Ru_0.94_Sn_0.06_ NPs/C and Ru_0.87_Sn_0.13_ NPs/C to achieve a current density of 10 mA cm^−2^ were 43.41 and 33.19 mV, respectively, which are lower than those of monometallic Ru NPs/C (53.53 mV) and commercial Pt NPs/C (55.77 mV). The valence-band structures of the NPs investigated by hard X-ray photoelectron spectroscopy demonstrated that the d-band centre of RuSn NPs shifted downward compared with that of Ru NPs. X-ray photoelectron spectroscopy and X-ray absorption near-edge structure analyses indicated that in the RuSn alloy NPs, charge transfer occurs from Sn to Ru, which was considered to result in a downward shift of the d-band centre in RuSn NPs and to regulate the adsorption energy of intermediate H_ads_ effectively, and thus enable the RuSn solid-solution alloy NPs to exhibit excellent HER catalytic properties.

## Abstract 2

Solid-solution RuSn alloy NPs were successfully synthesised for the first time and exhibited enhanced HER catalytic activity compared with Ru NPs and commercial Pt NPs under alkaline conditions, providing new insights for the design of catalysts.

## Graphical abstract

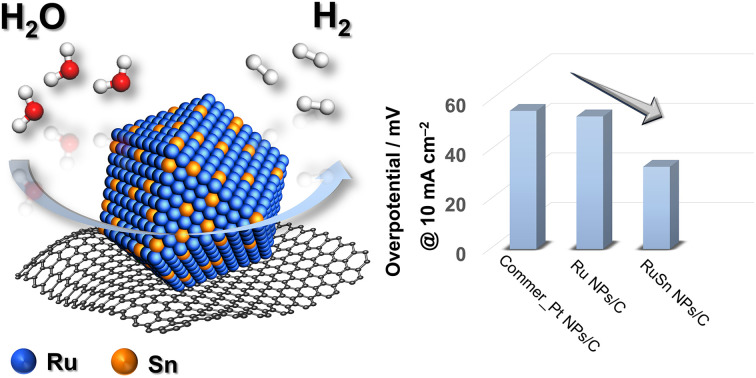

## Introduction

Platinum group metals (PGMs) have been extensively studied as catalysts in the automotive industry, petroleum refining, hydrogen production and electronics.^1–7^ Using PGMs as nanoparticles (NPs) is one of the effective ways to improve their catalytic performance, because the high surface-to-volume ratio and quantum size effect can be achieved.^8,9^ Alloying, which means mixing the metal constituents at the atomic level, is another effective strategy to improve the catalytic properties of PGMs. The electronic states and properties of PGM alloys are affected by changes in the constituent elements and/or composition.^10–13^ Actually, it has been reported that PGM-based solid-solution alloy NPs showed excellent catalytic performance, such as RuPd,^14^ RuIr,^15^ PtPd,^16^ RuPt,^17^ RuRh^18^ and PtIr^19^ alloy NPs. Currently, the constituent elements of reported PGM-based solid-solution alloys are mainly precious metals. However, PGMs are scarce and very expensive resources, and therefore alloying with non-noble metals would be an effective way of reducing PGM usage.

Tin (Sn) is a non-precious metal from group 14 of the periodic table of the elements. In recent years, alloys based on PGMs and Sn have been synthesised as catalysts.^20–24^ However, most reported PGM–Sn alloys have intermetallic structures with specific ordered atomic arrangements, and few reports have been reported on solid-solution alloy system of PGMs and Sn with disordered atomic arrangements. In contrast to intermetallic alloys, in which composition ratios cannot be flexibly changed, solid-solution alloys allow continuous control over metal composition ratios. Due to continuous composition control, solid-solution alloys possess the merits of precise tuning of the electronic structure as well as properties of PGM alloys. Thus, solid-solution alloy NPs with a continuously fine-tunable electronic structure might be fascinating materials for catalytic applications closely related to adsorption energy.^25^

In this work, we have focused on the Ru–Sn system. Although RuSn alloy NPs have the potential to be highly efficient catalysts, synthesising solid-solution RuSn alloy NPs remains challenges. There are three intermetallic compound phases in the bulk Ru–Sn system (Ru_2_Sn_3_, RuSn_2_, and Ru_3_Sn_7_).^26^ Therefore, the ratio of Ru/Sn is fixed to these three points and cannot be freely controlled. Because the electronic properties of Ru (a d-block metal) and Sn (a p-block metal) are significantly different, they tend to form intermetallic compounds with ordered structures, and therefore, RuSn solid-solution alloys with disordered structures are difficult to obtain.^27^ In addition, the large difference in redox potentials between Ru^3+^/Ru^0^ (0.35 V *vs.* the standard hydrogen electrode, SHE)^28^ and Sn^2+^/Sn^0^ (−0.14 V *vs.* SHE),^29^ making it difficult to achieve the obtainment of alloy NPs. Furthermore, Ru has hexagonal close-packed (hcp) structure as the most stable crystal structure, and Sn has cubic or tetragonal structure as the most stable structure. Their different crystal structures also make the synthesis of solid-solution RuSn alloy NPs challenging.^30,31^

**Scheme 1:**
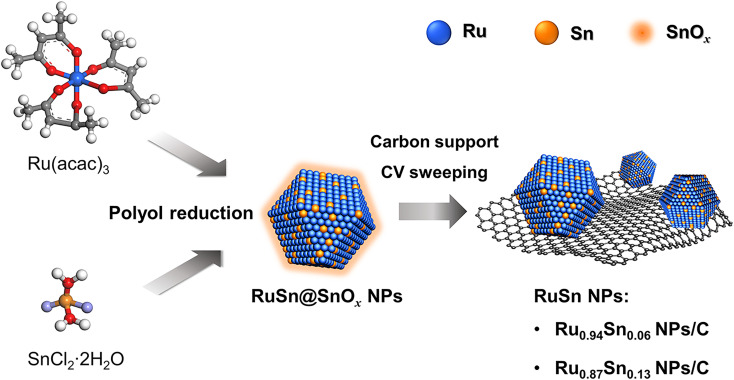
Synthesis of RuSn solid-solution alloy NPs from cyclic voltammetry sweeping of RuSn@SnO_*x*_ NPs.

Herein, the novel RuSn solid-solution alloy NPs were successfully synthesised *via* electrochemical cyclic voltammetry (CV) sweeping of pre-synthesised RuSn@SnO_*x*_ NPs (Scheme 1). The RuSn alloy NPs exhibited enhanced hydrogen evolution reaction (HER) performance compared with monometallic Ru NPs. Hard X-ray photoelectron spectroscopy (HAXPES) showed that RuSn alloy NPs have a deeper d-band centre in comparison to monometallic Ru NPs. X-ray photoelectron spectroscopy (XPS) and X-ray absorption spectroscopy revealed the charge transfer from Sn to Ru in RuSn alloy NPs. It is considered that the charge transfer from Sn to Ru results in a downward shift of the d-band centre in RuSn alloy NPs relative to Ru NPs, which leads to a weakened bond strength between the metal and the intermediate H_ads_, thus enabling the RuSn alloy NPs to exhibit excellent HER catalytic properties.

## Results and discussion

RuSn solid-solution alloy NPs were obtained from CV cleaning of the pre-synthesised RuSn solid-solution alloy NPs surrounded by Sn oxide species (RuSn@SnO_*x*_ NPs) (see the details in the ESI†). A polyol reduction method was used for the synthesis of RuSn@SnO_*x*_ NPs. Transmission electron microscopy (TEM), high-angle annular dark-field scanning transmission electron microscopy (HAADF-STEM), energy-dispersive X-ray spectroscopy (EDX) maps and XPS were conducted to characterise the synthesised RuSn@SnO_*x*_ NPs (Fig. S1–S4 and Table S1†). The synthesised NPs were confirmed to be RuSn solid-solution alloy NPs surrounded by Sn oxide species. By controlling the ratios of the Ru and Sn precursors, RuSn@SnO_*x*_ NPs with different metal composition ratios were achieved.

**Fig. 1:**
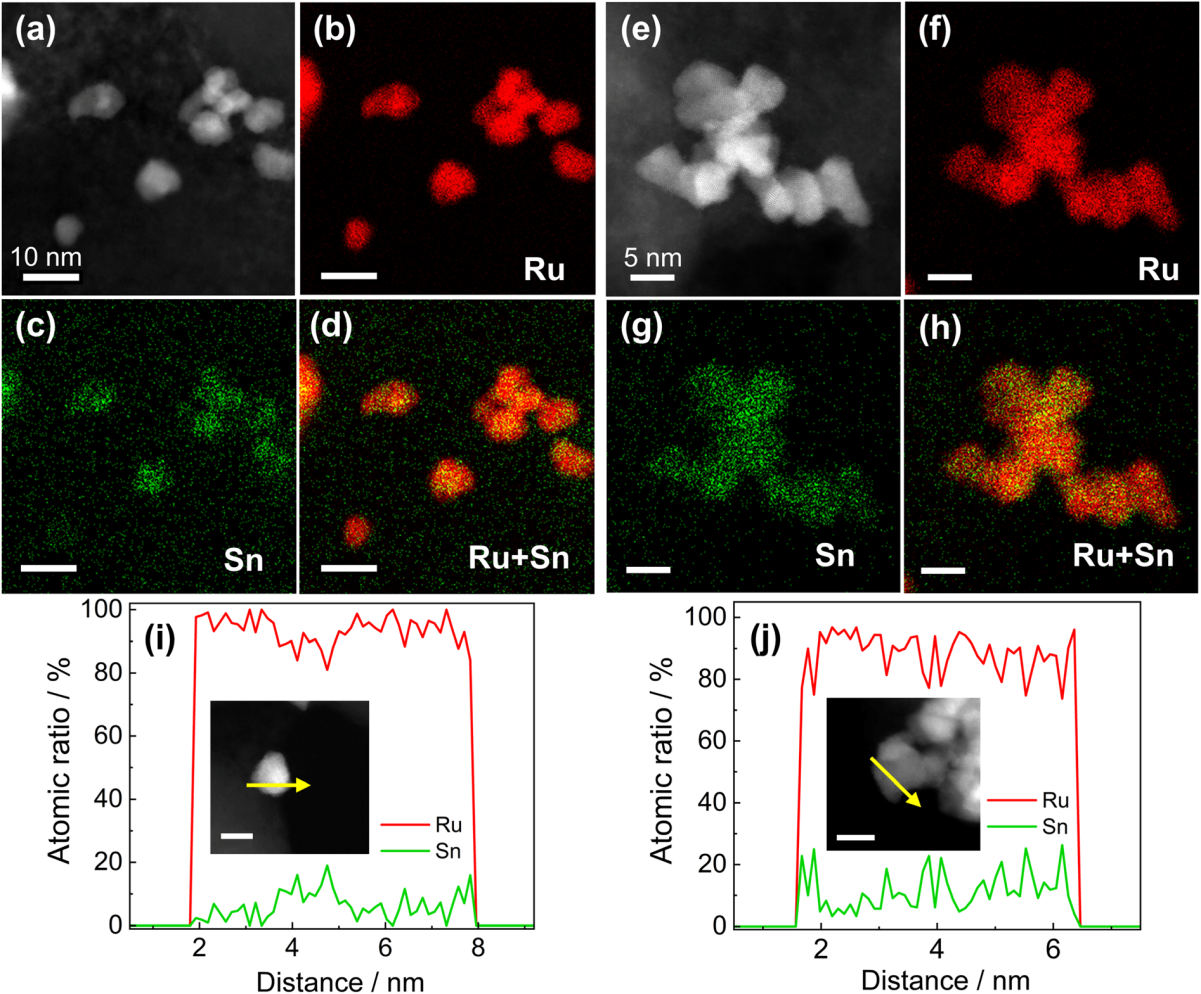
(a) HAADF-STEM image of Ru_0.94_Sn_0.06_ NPs/C. STEM-EDX elemental mapping of Ru_0.94_Sn_0.06_ NPs/C showing (b) Ru (L-line), (c) Sn (L-line) and (d) overlay images. (e) HAADF-STEM image of Ru_0.87_Sn_0.13_ NPs/C. STEM-EDX elemental mapping of Ru_0.87_Sn_0.13_ NPs/C showing (f) Ru (L-line), (g) Sn (L-line) and (h) overlay images. EDX line scan profiles of (i) Ru_0.94_Sn_0.06_ NPs/C and (j) Ru_0.87_Sn_0.13_ NPs/C. The arrow in the inset indicates the direction of the line scan. The scale bar in the inset is 5 nm.

To remove the surface Sn oxides, the RuSn@SnO_*x*_ NPs were first loaded on carbon, and then electrochemical CV sweeping was performed in an Ar-saturated 1.0 M KOH electrolyte with a rate of 500 mV s^−1^ in the potential range from 0.05 to 0.40 V (*vs.* reversible hydrogen electrode, RHE) for several hundred cycles until the obtained CV curves were stable. The HAADF-STEM images, corresponding EDX maps and line scan profiles indicated SnO_*x*_ was removed from the surface of RuSn@SnO_*x*_ NPs, and the Ru and Sn elements were randomly and uniformly distributed in the NPs (Fig. 1a–h, S5 and S6†). These results confirmed that solid-solution RuSn alloy NPs were successfully obtained. The EDX line scan analysis shows the atomic ratios of Sn in RuSn NPs to be 6% and 12% after CV sweeping (Fig. 1i and j). According to the X-ray fluorescence (XRF) results, the atomic ratios of Ru : Sn were 0.94 : 0.06 (Ru_0.94_Sn_0.06_ NPs) and 0.87 : 0.13 (Ru_0.87_Sn_0.13_ NPs), respectively, which is in agreement with the results of EDX line scan. The particle sizes of the CV-cleaned RuSn alloy NPs were shown by TEM to be 4.9 ± 1.5 and 5.0 ± 1.7 nm for Ru_0.94_Sn_0.06_ NPs and Ru_0.87_Sn_0.13_ NPs, respectively (Fig. S7†). The mean diameters of the NPs were averaged from 200 particles.

**Fig. 2:**
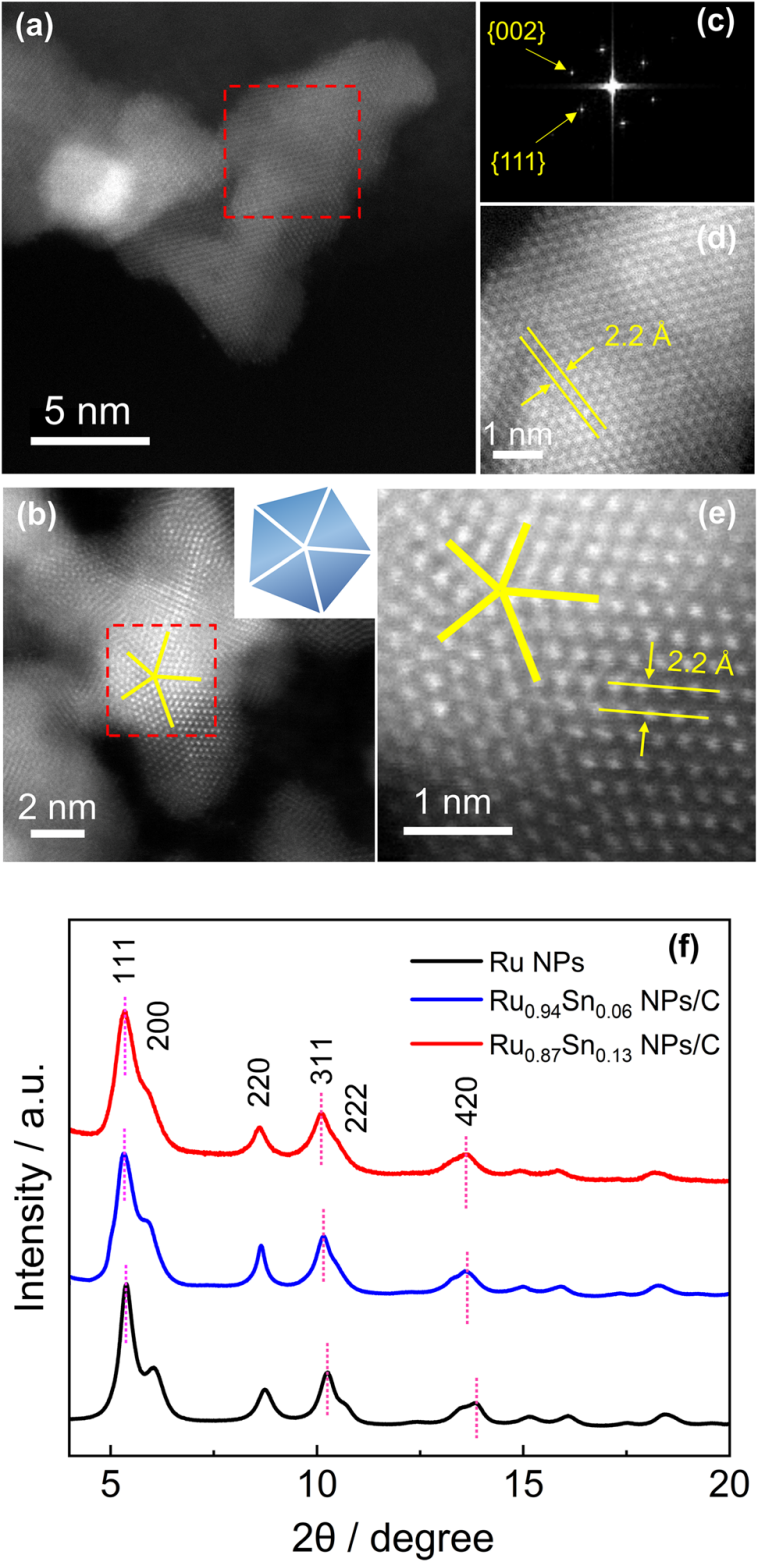
Atomic-resolution HAADF-STEM images of (a) Ru_0.94_Sn_0.06_ NPs/C and (b) Ru_0.87_Sn_0.13_ NPs/C. (c) FFT pattern of (a). (d) Expanded image of the red dashed square region in (a). (e) Expanded image of the red dashed square region in (b). (f) Synchrotron XRD patterns of Ru NPs, Ru_0.94_Sn_0.06_ NPs/C and Ru_0.87_Sn_0.13_ NPs/C. The radiation wavelength is 0.2069(1) Å.

The atomic arrangements of Ru_0.94_Sn_0.06_ NPs and Ru_0.87_Sn_0.13_ NPs were characterised by HAADF-STEM (Fig. 2a and b). The fast Fourier transform (FFT) pattern of Ru_0.94_Sn_0.06_ NPs revealed a face-centred cubic (fcc) crystal nature (Fig. 2c). From the FFT pattern, the calculated *d*-spacings were estimated to be 1.9 and 2.2 Å for the {002} and {111} planes, respectively. Fig. 2d shows the clear atomic arrangements, with measured lattice spacing of 2.2 Å, which is typical of the fcc lattice observed from the 1̄10 direction.^32^ The HAADF-STEM image of Ru_0.87_Sn_0.13_ NPs shows a five-fold symmetric twinned nanoparticle with a decahedral structure consisting of five tetrahedra (Fig. 2b), indicating the formation of the fcc structure.^33–35^ The enlarged STEM image exhibited the atomic arrangement of the NP with a lattice spacing of 2.2 Å, further indicating a typical of fcc structure viewed along the [1̄10] direction (Fig. 2e).

Synchrotron X-ray diffraction (XRD) was conducted to investigate the crystal structures of the CV-cleaned RuSn NPs. Monometallic Ru NPs were also synthesised and characterised for comparison (Fig. S8†). The XRD of the synthesised Ru NPs exhibited a single fcc diffraction pattern (Fig. 2f). It is well known that the most stable crystal structure of bulk Ru is hcp structure, but when the size is reduced to nanometer order, fcc Ru NPs can be obtained.^35^ From Rietveld refinement, the obtained lattice parameter *a* of the Ru NPs was 3.828(3) Å (Fig. S9a†), which is in agreement with the ideal fcc lattice parameter (3.82 Å) calculated from the hcp lattice parameter, by √2 × *a*_hcp_.^36^ The XRD peaks of the RuSn NPs shifted towards a lower angle than those of the monometallic Ru NPs (Fig. 2f). In addition, the peak positions of Ru_0.87_Sn_0.13_ NPs were lower than those of Ru_0.94_Sn_0.06_ NPs. These results suggested that RuSn solid-solution alloy NPs were successfully formed. The XRD diffraction patterns of the RuSn alloy NPs after CV cleaning were identical to those before CV cleaning, which implies the crystal structure of the NPs was not altered by CV scanning (Fig. S9b†), and metallic Sn atoms were still in the lattice of RuSn alloy NPs. The Rietveld refinement analysis revealed that the Ru_0.94_Sn_0.06_ NPs consisted of a major fcc phase (89.1%) and a minor hcp phase (10.9%) (Fig. S10a†). For fcc component, the obtained lattice constant was 3.861(3) Å. And for hcp component, the obtained lattice constants were 2.713(8) and 4.290(2) Å for *a*_hcp_ and *c*_hcp,_ respectively. The Rietveld refinement revealed a single fcc pattern for Ru_0.87_Sn_0.13_ NPs with an obtained lattice constant *a* of 3.899(8) Å (Fig. S10b†). The lattice parameter *a*_fcc_ for the NPs increased with an increasing proportion of Sn, which implies successful alloying of Sn with Ru in RuSn NPs. The lattice parameters for the hcp structure in Ru_0.94_Sn_0.06_ NPs were also larger than those of the value for hcp Ru (*a* = 2.70 Å, *c* = 4.27 Å),^37^ indicating that the hcp component of Ru_0.94_Sn_0.06_ NPs also forms a solid-solution alloy. In addition, the calculated fcc lattice spacings of the {111}_fcc_ planes of Ru_0.94_Sn_0.06_ NPs and Ru_0.87_Sn_0.13_ NPs were 2.2 Å, which are in agreement with the HAADF-STEM results. The synchrotron XRD results indicate that solid-solution RuSn alloy NPs with different composition ratios were successfully synthesised, which could not be achieved in intermetallic RuSn alloy NPs reported previously.^27^

**Fig. 3:**
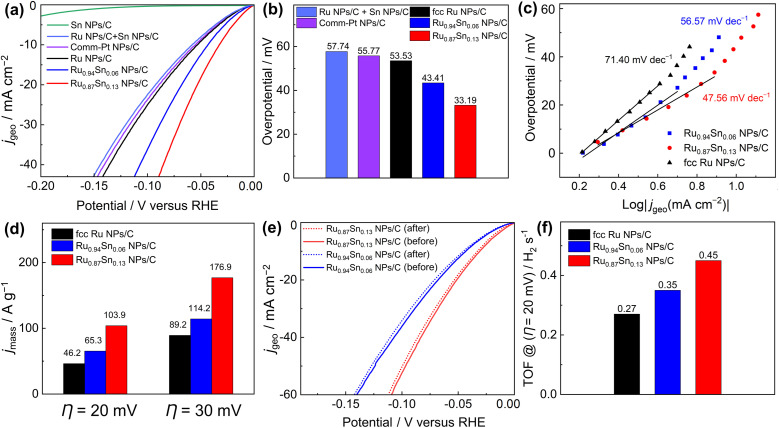
HER catalytic performance in Ar-saturated 1.0 M KOH. (a) Polarisation curves of Sn NPs/C, Ru NPs/C + Sn NPs/C, commercial Pt NPs/C, fcc Ru NPs/C, Ru_0.94_Sn_0.06_ NPs/C and Ru_0.87_Sn_0.13_ NPs/C. (b) Overpotentials of Ru NPs/C + Sn NPs/C, commercial Pt NPs/C, fcc Ru NPs/C, Ru_0.94_Sn_0.06_ NPs/C and Ru_0.87_Sn_0.13_ NPs/C. (c) Steady-state Tafel slopes of fcc Ru NPs/C, Ru_0.94_Sn_0.06_ NPs/C and Ru_0.87_Sn_0.13_ NPs/C. (d) Mass activities (at *η* of 20 and 30 mV) of fcc Ru NPs/C, Ru_0.94_Sn_0.06_ NPs/C and Ru_0.87_Sn_0.13_ NPs/C. (e) Polarisation curves of Ru_0.94_Sn_0.06_ NPs/C and Ru_0.87_Sn_0.13_ NPs/C before and after the durability test. (f) TOF values (at *η* of 20 mV) of fcc Ru NPs/C, Ru_0.94_Sn_0.06_ NPs/C and Ru_0.87_Sn_0.13_ NPs/C.

The HER catalytic performances of the carbon-supported NPs were recorded with a standard three-electrode system in an Ar-saturated 1.0 M KOH electrolyte with a pH of 14.0.^38^ Linear sweep voltammetry curves showed that the overpotentials of Ru_0.94_Sn_0.06_ NPs/C and Ru_0.87_Sn_0.13_ NPs/C at the current density of 10 mA cm^−2^ were 43.41 and 33.19 mV, respectively, which are lower than those of fcc Ru NPs (53.53 mV^−1^) and commercial Pt NPs/C (55.77 mV^−1^) (Fig. 3a and b). The HER properties of carbon black (Vulcan XC-72R) were also evaluated (Fig. S11†). The carbon black did not exhibit HER catalytic properties. The results showed that carbon black is inert for the HER catalytic reaction. To investigate the origin of the improved HER electrocatalytic activity in the Ru_0.94_Sn_0.06_ NPs and Ru_0.87_Sn_0.13_ NPs, we synthesised monometallic Sn NPs (Fig. S12†) and examined their HER activity. Monometallic Sn NPs/C did not demonstrate HER catalytic properties. Furthermore, physically mixed Ru NPs and Sn NPs were measured for HER catalytic performance (the Ru NPs and Sn NPs were mixed at an atomic ratio of 0.87 : 0.13), which exhibited a similar catalytic property to that of monometallic Ru NPs/C. These results indicate that Ru NPs performs a critical role in HER catalysis, and physical mixing with Sn NPs could not improve the catalytic performance of Ru NPs. Moreover, alloying with Sn could improve the HER performance of Ru NPs, and the catalytic performance was further enhanced with the increase in Sn content.

The steady-state Tafel slopes were measured by recording HER current at the 100 seconds of chronoamperometry responses at various potentials (−5 to −60 mV *vs.* RHE) with an interval of 5 mV.^39^ The steady-state Tafel slopes of Ru NPs/C, Ru_0.94_Sn_0.06_ NPs/C and Ru_0.87_Sn_0.13_ NPs/C were 71.40, 56.57 and 47.56 mV decade^−1^, respectively (Fig. 3c). The Tafel slope of RuSn NPs decreases with increasing Sn content, which indicates faster reaction kinetics of Ru_0.87_Sn_0.13_ NPs/C. The Tafel slopes of the NPs also imply a Volmer–Heyrovsky mechanism, and the Heyrovsky step is the rate-determining step of the reaction.^40–42^

As shown in Fig. 3d and S13,† the Ru_0.87_Sn_0.13_ NPs/C showed the highest mass activity for HER catalysis (103.9 A g^−1^ at an overpotential *η* of 20 mV; 176.9 A g^−1^ at 30 mV) compared with Ru_0.94_Sn_0.06_ NPs/C (65.3 and 114.2 A g^−1^ at 20 and 30 mV, respectively) and Ru NPs/C (46.2 and 89.2 A g^−1^ at 20 and 30 mV, respectively). Furthermore, Ru_0.87_Sn_0.13_ NPs/C and Ru_0.94_Sn_0.06_ NPs/C showed excellent durability in alkaline solutions with negligible degradation. The polarisation curves of Ru_0.87_Sn_0.13_ NPs/C and Ru_0.94_Sn_0.06_ NPs/C after the 10 h chronoamperometry measurement did not show noticeable degradation in comparison with that of monometallic Ru NPs/C (Fig. 3e, S14 and S15†). Powder XRD measurement was carried out on the Ru_0.94_Sn_0.06_ NPs and Ru_0.87_Sn_0.13_ NPs after the 10 h chronoamperometry measurement, and the positions of the XRD diffraction peaks before and after the measurement were identical, and no diffraction peaks derived from the oxides were observed, indicating that the crystal structure was not obviously changed after the 10 h measurement (Fig. S16†). According to the XRF results, the atomic ratios of Ru to Sn in Ru_0.94_Sn_0.06_ NPs and Ru_0.87_Sn_0.13_ NPs after the stability test are 0.946 : 0.054 and 0.884 : 0.116, respectively. The results showed that there is only a slight reduction in the Sn content after the 10 h reaction, with most of the Sn content remaining in the alloy NPs. The XPS spectra of RuSn NPs after chronoamperometry measurement were also investigated (Fig. S17†). The Ru 3p and Sn 3d XPS spectra of RuSn NPs after the measurement displayed only XPS peaks for the metallic Ru and Sn, suggesting that the elements remain in the metallic state in the alloy NPs. The results showed that the crystal structure of RuSn NPs before and after the chronoamperometry measurement were identical and no significant oxidation of NPs was observed.

The turnover frequency (TOF) serves as an accurate description of the intrinsic activity of a catalyst. However, the TOF is difficult to measure directly, so it is typically inferred from the measurements of the electrochemically active surface area (ECSA) and active sites (*n*). The ECSA for the catalyst was calculated *via* the method of underpotential deposition of copper (Fig. S18†). The ECSA and number of active sites were evaluated to be similar for Ru_0.87_Sn_0.13_ NPs/C, Ru_0.94_Sn_0.06_ NPs/C and Ru NPs/C (Fig. S19†). At an overpotential of 20 mV, the TOF values of Ru_0.94_Sn_0.06_ NPs/C and Ru_0.87_Sn_0.13_ NPs/C were 0.35 and 0.45 H_2_ s^−1^, respectively, higher than the value for Ru NPs/C (0.27 H_2_ s^−1^) (Fig. 3f). The relatively lower overpotential and increased TOF value indicated that the RuSn solid-solution alloy NPs possess superior HER performance, which is one of the most promising Ru-based catalysts reported for HER under alkaline conditions (Table S2†).

**Fig. 4:**
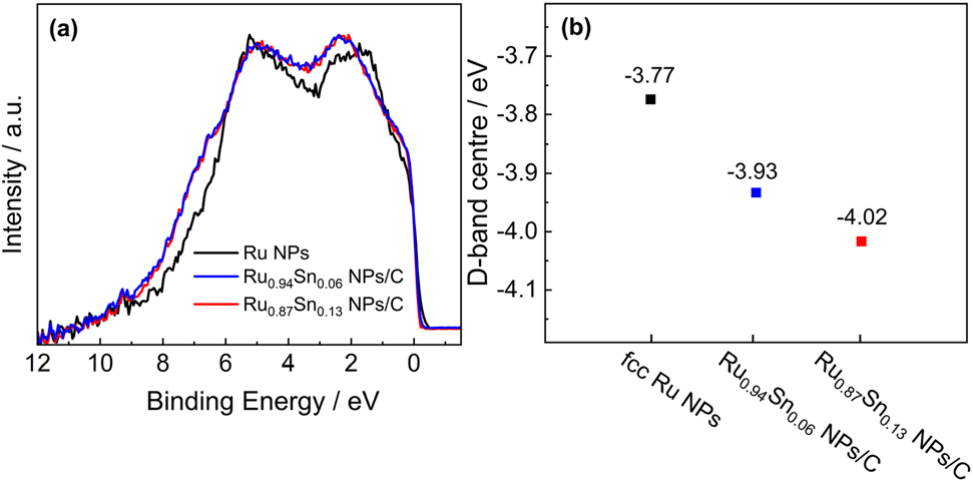
(a) VB HAXPES spectra of Ru NPs, Ru_0.94_Sn_0.06_ NPs/C and Ru_0.87_Sn_0.13_ NPs/C. (b) d-band centres of Ru NPs, Ru_0.94_Sn_0.06_ NPs/C and Ru_0.87_Sn_0.13_ NPs/C.

To elucidate the enhanced HER activity of RuSn solid-solution alloy NPs, HAXPES was carried out to investigate the electronic structures of Ru_0.94_Sn_0.06_ NPs, Ru_0.87_Sn_0.13_ NPs and Ru NPs. From the valence-band (VB) HAXPES spectra (Fig. 4a), we confirmed the systematic change in the density of states, which is caused by alloying Sn with Ru. The d-band centre, which is known as a useful descriptor for understanding the HER activity,^43–45^ was estimated from the VB HAXPES spectra. The estimated d-band centre of the Ru NPs was −3.77 eV. The d-band centre of RuSn alloy NPs continuously decreased with increasing Sn content, namely, −3.93 eV for Ru_0.94_Sn_0.06_ NPs and −4.02 eV for Ru_0.87_Sn_0.13_ NPs (Fig. 4b), which were deeper than that of Ru NPs. According to the d-band theory, the downward shift of the d-band centre will increase the filling of the anti-bonding states between the intermediate H_ads_ and the metal, which leads to a weakened hydrogen adsorption energy.^46–50^ Considering that the adsorption energy of monometallic Ru is too strong, the weakened adsorption energy derived from alloying with Sn might contribute to the enhancement of the HER activity in RuSn alloy NPs.

**Fig. 5:**
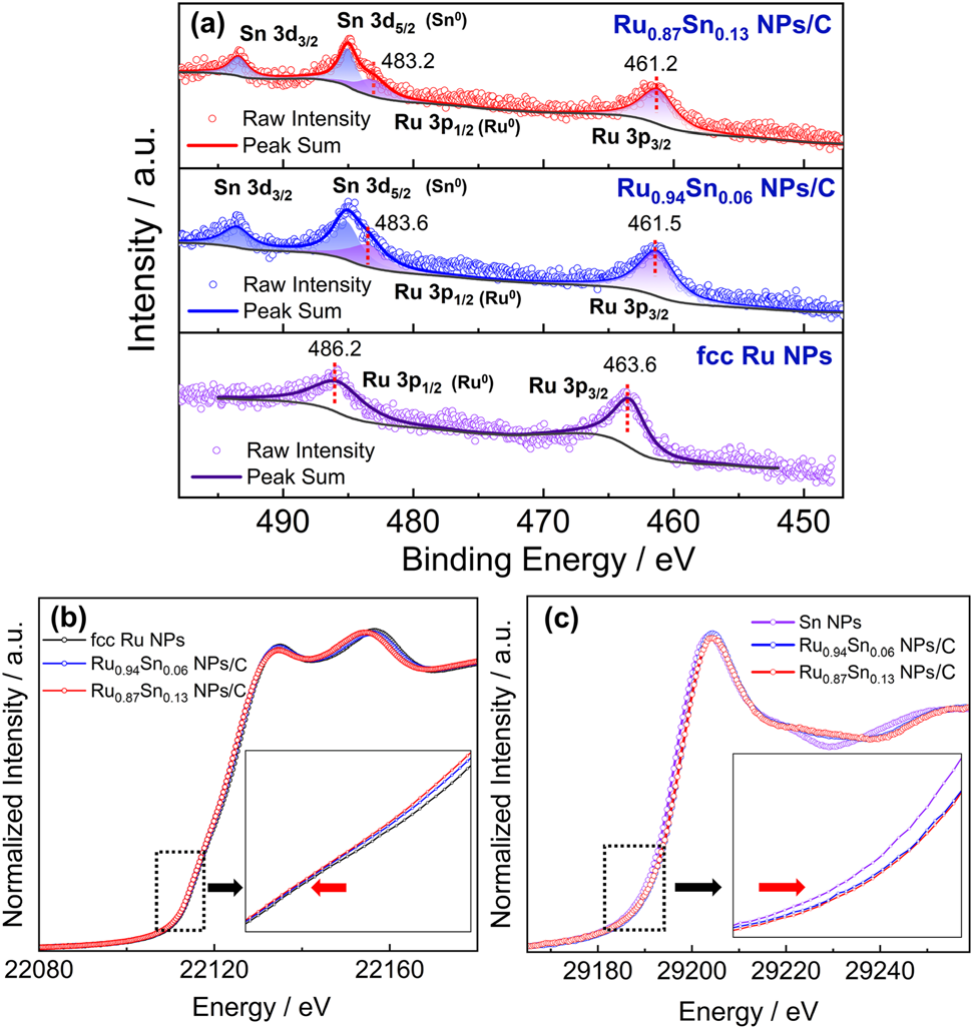
(a) Ru 3p XPS spectra of fcc Ru NPs, Ru_0.94_Sn_0.06_ NPs/C and Ru_0.87_Sn_0.13_ NPs/C. The black curves serve as the background files. (b) Ru K-edge XANES spectra of fcc Ru NPs, Ru_0.94_Sn_0.06_ NPs/C and Ru_0.87_Sn_0.13_ NPs/C. (c) Sn K-edge XANES spectra of Sn NPs, Ru_0.94_Sn_0.06_ NPs/C and Ru_0.87_Sn_0.13_ NPs/C.

The XPS spectra of Ru NPs, Ru_0.94_Sn_0.06_ NPs and Ru_0.87_Sn_0.13_ NPs were used to investigate the change in the electronic interaction induced by alloying Ru and Sn at the atomic level (Fig. 5a). The Ru 3p XPS spectrum of Ru NPs exhibited two asymmetric peaks at the binding energies of 463.6 and 486.2 eV, which were attributed to 3p_3/2_ and 3p_1/2_ of metallic Ru.^51,52^ The Ru 3p XPS peaks of RuSn alloy NPs were noticeably negatively shifted relative to those of monometallic Ru NPs. The negatively shifted Ru 3p_3/2_ and Ru 3p_1/2_ peaks in RuSn NPs indicate the charge transfer from Sn to Ru, making the Ru atoms negatively charged in RuSn alloy NPs, derived from alloying Ru and Sn at the atomic level.

This phenomenon was further confirmed by X-ray absorption fine structure analysis. The Ru K-edge X-ray absorption near-edge structure (XANES) spectra of Ru_0.87_Sn_0.13_ NPs, Ru_0.94_Sn_0.06_ NPs and Ru NPs are shown in Fig. 5b. The absorption edge gradually shifted to the lower energy side with the increase in Sn content. This indicates that Ru in the RuSn alloy NPs became negatively charged with Sn composition.^53,54^ The absorption K-edge of Sn gradually shifted to the higher energy side with increasing Sn content in the alloy NPs, which suggests a reduction in the electron density of Sn in RuSn alloy NPs (Fig. 5c). These results were consistent with the XPS results.

It has been reported that when the p-block metal is alloyed with the d-block metal, the charge transfer will occur from a p-block metal to a d-block metal, and the d-band centre of the transition metal will shift downwards.^55–58^ For example, in the Pt–Sn alloy system, the d-band centre of the PtSn alloy is deeper than that of the monometallic Pt due to the charge transfer from Sn to Pt, indicating the role of Sn as a modifier of the Pt electronic band structure.^58^ In this work, it was assumed that the charge transfer from Sn to Ru modifies the electronic structure of Ru in RuSn alloy NPs, which deepens the d-band centre of the RuSn alloy NPs compared with monometallic Ru NPs. This weakens the interaction between adsorbed H_ads_ and the metal (RuSn) surface, thus facilitating the desorption of H_ads_ from the surface of RuSn alloy NPs.^59–61^ Therefore, the alloying of Sn with Ru enables the enhancement of the HER activity of Ru NPs.

## Conclusions

In summary, for the first time, novel solid-solution RuSn alloy NPs were synthesised by electrochemical CV sweeping of RuSn@SnO_*x*_ NPs. STEM-EDX mapping and synchrotron XRD revealed that the Ru and Sn elements are randomly and uniformly distributed in RuSn alloy NPs. The novel RuSn alloy NPs demonstrated enhanced HER activity under alkaline conditions, which is superior to that of monometallic Ru NPs and commercial Pt NPs/C. VB HAXPES spectra showed that the d-band centre of RuSn solid-solution alloy NPs shifted downwards compared with that of Ru NPs. XPS and XANES revealed the charge transfer from Sn to Ru in RuSn alloy NPs. It is considered that the charge transfer from Sn to Ru results in the d-band centre of RuSn alloy NPs shifted downwards, which modulates the adsorption energy of H_ads_ and enhances the HER activity. This research provides a valuable perspective for the development of higher-performance and lower-cost catalysts, not only for the Ru–Sn system, but also for other d-block and p-block metal systems.

## Data availability

The datasets supporting this article have been uploaded as part of the ESI.†

## Author contributions

X. Z., M. M., K. K. and H. K. conceived the research and designed the experiments; X. Z. synthesised the materials; T. Y., T. T. and Y. M. conducted the STEM characterisation; S. K. and Y. K. carried out the synchrotron XRD test; O. S. and O. S. carried out the HAXPES measurement; T. I. performed the XANES measurement; X. Z. and M. M. wrote the manuscript; all authors revised the manuscript; all authors contributed to the discussion of results and commented on the manuscript.

## Conflicts of interest

There are no conflicts to declare.

## Supplementary Material

SC-015-D3SC06786F-s001
