# Supplementary material for: First synthesis of RuSn solid-solution alloy nanoparticles and their enhanced hydrogen evolution reaction activity
Source: Chem Sci. 2024 Apr 16;15(20):7560–7. doi: 10.1039/d3sc06786f (PMC11110130; doi:10.1039/d3sc06786f)
Supplement: SC-015-D3SC06786F-s001 [file SC-015-D3SC06786F-s001.pdf]

Electronic Supplementary Material (ESI) for Chemical Science.
This journal is © The Royal Society of Chemistry 2024

Support Information

First synthesis of RuSn solid-solution alloy nanoparticles and their enhanced hydrogen evolution reaction activity

Xin Zhou,[a] Megumi Mukoyoshi,[a*] Kohei Kusada,[a,b,c] Tomokazu Yamamoto,[d] Takaaki Toriyama,[d] Yasukazu Murakami,[d,e] Shogo Kawaguchi,[f] Yoshiki Kubota,[g] Okkyun Seo,[f,h] Osami Sakata,[f,h] Toshiaki Ina,[f] and Hiroshi Kitagawa[a*]

[a.] Division of Chemistry, Graduate School of Science, Kyoto University, Kitashirakawa-Oiwakecho, Sakyo-ku, Kyoto 606-8502, Japan.

[b.] The HAKUBI Center for Advanced Research, Kyoto University, Kitashirakawa-Oiwakecho, Sakyo-ku, Kyoto 606-8502, Japan.

[c.] JST-PRESTO, Honcho 4-1-8, Kawaguchi, Saitama 332-0012, Japan.

[d.] The Ultramicroscopy Research Center, Kyushu University, 744 Motooka, Nishi-ku, Fukuoka 819-0395, Japan.

[e.] Department of Applied Quantum Physics and Nuclear Engineering, Kyushu University, 744 Motooka, Nishi-ku, Fukuoka 819-0395, Japan.

[f.] Center for Synchrotron Radiation Research, Japan Synchrotron Radiation Research Institute (JASRI) SPring-8, 1-1-1 Kouto, Sayo-cho, Sayo-gun, Hyogo 679-5198, Japan.

[g.] Department of Physics, Graduate School of Science, Osaka Metropolitan University, Sakai, Osaka 599-8531, Japan.

[h.] Research Network and Facility Services Division, National Institute for Materials Science (NIMS), 1-1-1 Kouto, Sayo-cho, Sayo-gun, Hyogo 679-5148, Japan.

*Corresponding authors:
Megumi Mukoyoshi (mukoyoshi@ssc.kuchem.kyoto-u.ac.jp);
Hiroshi Kitagawa (kitagawa@kuchem.kyoto-u.ac.jp)

Table of Contents

Experimental Details 3

Figure S1. TEM images of RuSn@SnO$_x$ NPs..9

Figure S2. HAADF-STEM images of RuSn@SnO$_x$ NPs 10

Figure S3. XPS spectra of RuSn@SnO$_x$ NPs...11

Table S1. XPS peak area of Sn 3d$_{5/2}$ in RuSn@SnO$_x$ NPs......................................11

Figure S4. O 1s XPS spectra 12

Figure S5. EDX line scan profiles of Ru$_{0.94}$Sn$_{0.06}$ NPs/C 13

Figure S6. EDX line scan profiles of Ru$_{0.87}$Sn$_{0.13}$ NPs/C 14

Figure S7. TEM images of RuSn@SnO$_x$ NPs...15

Figure S8. TEM image of Ru NPs 16

Figure S9. Synchrotron XRD of Ru NPs, RuSn@SnO$_x$ NPs and RuSn NPs/C..............17

Figure S10. Rietveld refinement analysis of RuSn NPs/C..18

Figure S11. HER catalytic performance of carbon black...19

Figure S12. TEM image and powder XRD pattern of Sn NPs...................................20

Figure S13. HER mass catalytic activities of the NPs...21

Figure S14. The chronoamperometry curves of RuSn NPs/C...................................22

Figure S15. The chronoamperometry curve of Ru NPs/C...23

Figure S16. Powder XRD of RuSn NPs/C after the stability test 24

Figure S17. XPS of RuSn NPs/C after the stability test 25

Figure S18. CV curves for ECSA measurement of the NPs......................................26

Figure S19. Number of active sites and ECSA of the NPs..27

Table S2. Comparison of Ru-based catalysts with high HER activity........................28

References...30

Chemicals

Triethylene glycol (TEG) and poly(N-vinyl-2-pyrrolidone) (PVP, K30, MW ≈ 40 000) were purchased from Wako Pure Chemical Industries, Ltd. (Japan). Ruthenium (III) acetylacetonate ($Ru(C_5H_7O_2)_3$, $Ru(acac)_3$), tin (II) chloride dihydrate ($SnCl_2{\cdot}2H_2O$), tin (II) acetate ($Sn(C_2H_3O_2)_2$), sodium borohydride ($NaBH_4$) and 1,5-pentanediol ($HO(CH_2)_5OH$) were purchased from Sigma-Aldrich Chemical Co. (USA). Commercial platinum 20 wt% on carbon (Commercial Pt NPs/C) was purchased from Johnson Matthey Co., Ltd. (UK). All reagents were used as received without further purification.

General characterisations

Transmission electron microscopy (TEM) images were obtained by a Hitachi HT7700 instrument at 100 kV.

High-angle annular dark-field scanning transmission electron microscopy (HAADF-STEM) images and energy-dispersive X-ray (EDX) spectra were acquired using a JEOL JEM-ARM200CF STEM instrument, equipped with an aberration corrector operated at 120 kV at Kyushu University, Japan.

X-ray fluorescence (XRF) was measured with a Rigaku ZSX Primus IV instrument.

Synchrotron X-ray diffraction (XRD) measurements were conducted on the BL13XU beamline at SPring-8, Japan, with multiple Lambda 750 K detectors. The radiation wavelength (λ) was 0.2069(1) Å. All XRD patterns were obtained in 0.005° steps.

Powder X-ray diffraction (PXRD) measurements were performed with a Rigaku MiniFlex 600 equipped with a Cu Kα radiation source (λ = 1.5418(2) Å).

Rietveld refinements were carried out with the TOPAS3 software developed by Bruker AXS GmbH (Karlsruhe, Germany).

X-ray photoelectron spectroscopy (XPS) results were recorded with a Shimadzu ESCA-3400 X-ray photoelectron spectrometer using a Mg Kα anode. The binding energy was corrected based on the C (1s) peak at 284.6 eV.

Valence band hard X-ray photoelectron spectroscopy (VB HAXPES) spectra were measured at the beamline BL46XU at SPring-8, Japan. The incident X-ray energy for HAXPES measurements was fixed to 7.94 keV. By measuring the Fermi cut-off of an evaporated Au thin film, the total energy resolution was estimated to be 0.2 eV. The binding energy is considered the Fermi level of the Au thin film. To remove the background, the VB HAXPES spectra of carbon powder were also measured. All the VB spectra were normalized according to the spectral area at the binding energy of 12.0 to −1.5 eV.

X-ray absorption near-edge structure (XANES) analysis was conducted on the BL01B1

beamline at SPring-8 (Japan) with synchrotron radiation sources and analysed by Athena software.

Synthesis of Ru nanoparticles (NPs)

In a typical synthesis, 80.0 mL of the TEG solution, which contained 222.0 mg PVP, was preheated to 100℃ and vacuumed for 1 h. TEG and PVP were used as a reducing agent and surfactant, respectively. Then, the above solution was heated to 230℃. 20.0 mL of the TEG solution with 0.2 mmol $Ru(acac)_3$ was added dropwise to the above preheated TEG solution with a speed of 0.2 mL/min. After the addition was completed, the solution was maintained at this temperature for a further 10 min. All reaction processes were carried out under a continuous Ar gas flow. Finally, the black powder was collected by washing with ethanol and ether, and centrifugation at 7800 rpm.

Synthesis of RuSn@SnO$_x$ NPs

The typical synthesis procedure was the same as that described above for Ru NPs. The precursor solution became a mixture of $Ru(acac)_3$ and $SnCl_2{\cdot}2H_2O$. Here, the total amount of the metal precursor was 0.2 mmol, and the molar ratios of the metal precursors were 8:2 and 9:1 for the syntheses of $Ru_{0.8}Sn_{0.2}$@SnO$_x$ NPs and $Ru_{0.9}Sn_{0.1}$@SnO$_x$ NPs, respectively. All reaction processes were carried out in a continuous Ar gas flow. Finally, the black powder was collected by washing with ethanol and ether, and centrifugation at 7800 rpm.

Characterisation of RuSn@SnO$_x$ NPs

TEM was used to determine the diameters of the synthesised RuSn@SnO$_x$ NPs, which were observed to be 5.1 ± 1.5 nm for $Ru_{0.9}Sn_{0.1}$@SnO$_x$ NPs and 5.3 ± 1.7 nm for $Ru_{0.8}Sn_{0.2}$@SnO$_x$ NPs (Fig. S1). The mean diameters of the NPs were averaged from 200 particles.

To confirm the mixing states of Ru and Sn elements in RuSn@SnO$_x$ NPs, HAADF-STEM analysis was performed. The STEM images and corresponding EDX maps revealed that the surface of the synthesised RuSn solid-solution alloy NPs was covered by excess Sn oxide species (Fig. S2). XPS was carried out to obtain information about the oxidation state of the metal elements in RuSn@SnO$_x$ NPs. For $Ru_{0.9}Sn_{0.1}$@SnO$_x$ NPs, the Sn 3d XPS spectrum has two peaks at binding energies (BEs) of 485.0 and 486.6 eV, which were attributed to $3d_{5/2}$ of metallic Sn and SnO, respectively (Fig. S3a and Table S1).[1] For

$Ru_{0.8}Sn_{0.2}@SnO_x$ NPs, the XPS spectrum of Sn $3d_{5/2}$ for metallic Sn and SnO was observed at BEs of 485.1 and 486.6 eV, respectively (Fig. S3b and Table S1). The XPS spectrum of Ru 3p for $RuSn@SnO_x$ NPs (Fig. S3a and b) exhibited only two peaks for metallic Ru, namely, 461.5 and 484.8 eV for $Ru_{0.9}Sn_{0.1}@SnO_x$ NPs, and 461.3 and 484.5 eV for $Ru_{0.8}Sn_{0.2}@SnO_x$ NPs, which were assigned to $3p_{3/2}$ and $3p_{1/2}$ of metallic Ru,[2] respectively. When the samples were subjected to 60 s of Ar etching, there were only peaks of metallic Sn and metallic Ru in the XPS spectra for $RuSn@SnO_x$ NPs (Fig. S3c and d). The XPS results indicated that the internal Ru and Sn were in a metallic state, and the surface Sn species were partially oxidised to Sn oxide species as $RuSn@SnO_x$. The O element of the $Ru_{0.9}Sn_{0.1}@SnO_x$ NPs and $Ru_{0.8}Sn_{0.2}@SnO_x$ NPs was homogeneously distributed in the NPs from the STEM EDX maps (Fig. S2e and j). The O 1s XPS spectra of $Ru_{0.9}Sn_{0.1}@SnO_x$ NPs and $Ru_{0.8}Sn_{0.2}@SnO_x$ NPs showed two peaks at BEs around 532.5 and 533.5 eV (Fig. S4a and b). In addition, the O 1s XPS spectra of carbon support, Ru NPs, $Ru_{0.94}Sn_{0.06}$ NPs and $Ru_{0.87}Sn_{0.13}$ NPs were also measured (Fig. S4c–f). All the samples showed the O 1s XPS peaks at BEs around 532.5 and 533.5 eV, which are related to the –OH and C=O groups, C–O and O–C=O groups, respectively.[3,4] The results indicate that O 1s XPS peaks of NPs originate from the oxygen-containing groups on the carbon black. It is difficult to distinguish the O 1s XPS peak of O in SnO_x from that of O in carbon support, but in the STEM EDX maps O element was noticeable around the NPs, indicating the presence of oxidation of the NPs.

According to the XRF results, the atomic ratios of Ru to Sn in $Ru_{0.9}Sn_{0.1}@SnO_x$ NPs and $Ru_{0.8}Sn_{0.2}@SnO_x$ NPs are 0.91:0.09 and 0.82:0.18, respectively. And after cyclic voltammetry (CV) sweeping to remove the surface oxides, the atomic ratios of Ru to Sn were 0.94:0.06 and 0.87:0.13 for $Ru_{0.94}Sn_{0.06}$ NPs and $Ru_{0.87}Sn_{0.13}$ NPs from the XRF, respectively. Thus, it could be obtained from the XRF results that $Ru_{0.9}Sn_{0.1}@SnO_x$ NPs could be interpreted as $Ru_{0.91}Sn_{0.06}@0.03SnO_x$ NPs and $Ru_{0.8}Sn_{0.2}@SnO_x$ NPs could be interpreted as $Ru_{0.82}Sn_{0.12}@0.06SnO_x$ NPs.

Synthesis of Sn NPs

A modified polyol process was used to synthesise Sn NPs.[5] $Sn(C_2H_3O_2)_2$ was used as the Sn precursor, $NaBH_4$ as the reducing agent, PVP as the surface stabilizer and 1,5-pentanediol as the solvent. First, 1.690 g of PVP and 0.144 g of $Sn(C_2H_3O_2)_2$ were dissolved in 50.0 mL of 1,5-pentanediol. The mixture was then heated to 160℃. Next, $NaBH_4$ (0.231 g dissolved in 10.0 mL of 1,5-pentanediol) was injected into the above

solution. During the reaction, an initial light-yellow solution sharply turned black, indicating the formation of Sn NPs. The solution was maintained at 160℃ for a further 20 min, and then cooled. All reaction processes were carried out in a continuous Ar gas flow. The powder was subsequently obtained by centrifugation at 7800 rpm and washed with ethanol several times.

Electrochemical measurements

The electrochemical process of the hydrogen evolution reaction (HER) was carried out and repeated three times using an electrochemical analyser (CHI760E, CH Instruments, USA) at room temperature. All measurements were performed in a three-electrode system in an Ar-saturated 1.0 M KOH electrolyte, with a rotating disk electrode (RDE, 5 mm diameter), Hg/HgO (in 1.0 M KOH) and a graphite rod as the working, reference and counter electrodes, respectively.

The catalyst samples were prepared by loading the as-synthesised NPs onto commercial carbon black (Vulcan XC-72R). Taking the preparation of the carbon-loaded Ru NPs catalyst with a metal percentage around 20 wt% as an example, 10.0 mg of Ru NPs and 40.0 mg of carbon black were dispersed in 40.0 mL of ethanol and then sonicated for 4 h. The final product was collected by centrifugation and dried overnight under vacuum conditions to obtain the carbon-loaded catalyst.

The catalyst ink was obtained as follows: 5.0 mg of the above carbon-loaded powder was dispersed into a mixture containing 1600.0 μL isopropanol, 300.0 μL water and 100.0 μL 5 wt% Nafion solution and sonicated for 2 h in an ice-water bath. The glassy carbon electrodes for electrochemical reactions were sequentially polished with diamond and Al_2O_3 solution. Hereafter, 10.0 μL of the above-obtained catalyst ink was dropped onto the electrode (5 mm diameter, 0.196 cm^2) and dried overnight. The mass loading density of RuSn NPs/C on a glassy carbon electrode is 0.1276 mg/cm^{-2}, which corresponds to the mass loading density of 0.0255 mg/cm^{-2} for RuSn NPs on a glassy carbon electrode.

The 1.0 M KOH electrolyte was bubbled under Ar for 40 min prior to the electrochemical process. The working electrode was first subjected to cyclic voltammetry (CV) sweeping at a voltage of 0.05~0.4 V (vs. RHE) with 500 mV s^{-1} for several hundred cycles until a stable CV curve was obtained.

The HER polarisation curves were recorded at a potential range of 0.05 to −0.2 V vs. RHE with a scanning speed of 5 mV s^{-1}. Chronoamperometry was applied to measure the long-

term stability of HER. All data were further used for the Ohmic drop correction at 85% compensation.

The underpotential deposition of copper (Cu-UPD) method was used to measure the electrochemical active surface area (ECSA) and the number of active sites (*n*), and the turnover frequency (TOF) of the catalyst was also calculated.[6–9]
The ECSA, n, and TOF were calculated by the following equations (1), (2) and (3), respectively:

$$ECSA = \frac{Q_{cu}}{0.42\ mC \cdot cm^{-2}} = \frac{\int_{E_1}^{E_2} I(E)d(E)}{v \cdot 0.42\ mC \cdot cm^{-2}} \quad (1)$$

$$n = \frac{Q_{cu}}{2F} \quad (2)$$

$$TOF = \frac{I}{2Fn} \quad (3)$$

where Q_{Cu} is the voltammetry charge of Cu-UPD after deducting the blank CV profile in pure Ar-saturated 0.5 M H_2SO_4, I is the current (A) of the linear sweep measurement and 0.42 mC cm^{-2} is the value of the charge consumed to form a Cu-UPD monolayer on the active metal sites. E_1 and E_2 are the lower and upper limits of the Cu-UPD peak, respectively. v is the scan rate of the CV curve. F is the Faraday constant (96,485 C mol^{-1}). A factor of 2 in equations (2) and equation (3) is based on the fact that two electrons are necessary to form one hydrogen molecule.

The Cu-UPD test was performed at room temperature. First, several hundred cycles of CV sweeping were performed on the electrode at a high speed of 500 mV s^{-1} in the range of 0.05~1.00 V vs. RHE in Ar-saturated 0.5 M H_2SO_4 to remove surface oxides. Then, several CV fine scans were recorded as the background in the range of 0.05~1.00 V at a rate of 10 mV s^{-1} in the Ar-saturated 0.5 M H_2SO_4. Subsequently, the electrolyte was changed to Ar-saturated 0.5 M H_2SO_4 + 5 mM $CuSO_4$. The electrode was first polarised for 100 s at a certain deposition potential to form a Cu monolayer. Finally, the Cu-UPD layer was stripped by CV scanning at a speed of 10 mV s^{-1} from the fixed potential to the upper potential.

Valence-band hard X-ray photoelectron spectroscopy (VB HAXPES)

The d-band centre of the NPs is calculated according to the equation (4):

$$\overline{E}_{d-band} = -\frac{\int E \times DOS(E)dE}{\int DOS(E)dE} \quad (4)$$

where E is binding energy and DOS(E) is the density of states (DOS) of the occupied d states. Here, we used $I(E)$, the intensity of the observed VB HAXPES spectrum, to represent DOS(E), and the centre of gravity of the VB HAXPES spectrum is defined as the d-band centre. The observed d-band centres of the NPs were obtained using the equation (5):

$$\overline{E}_{d-band}^{obs} = -\frac{\int E \times I(E)dE}{\int I(E)dE} \tag{5}$$

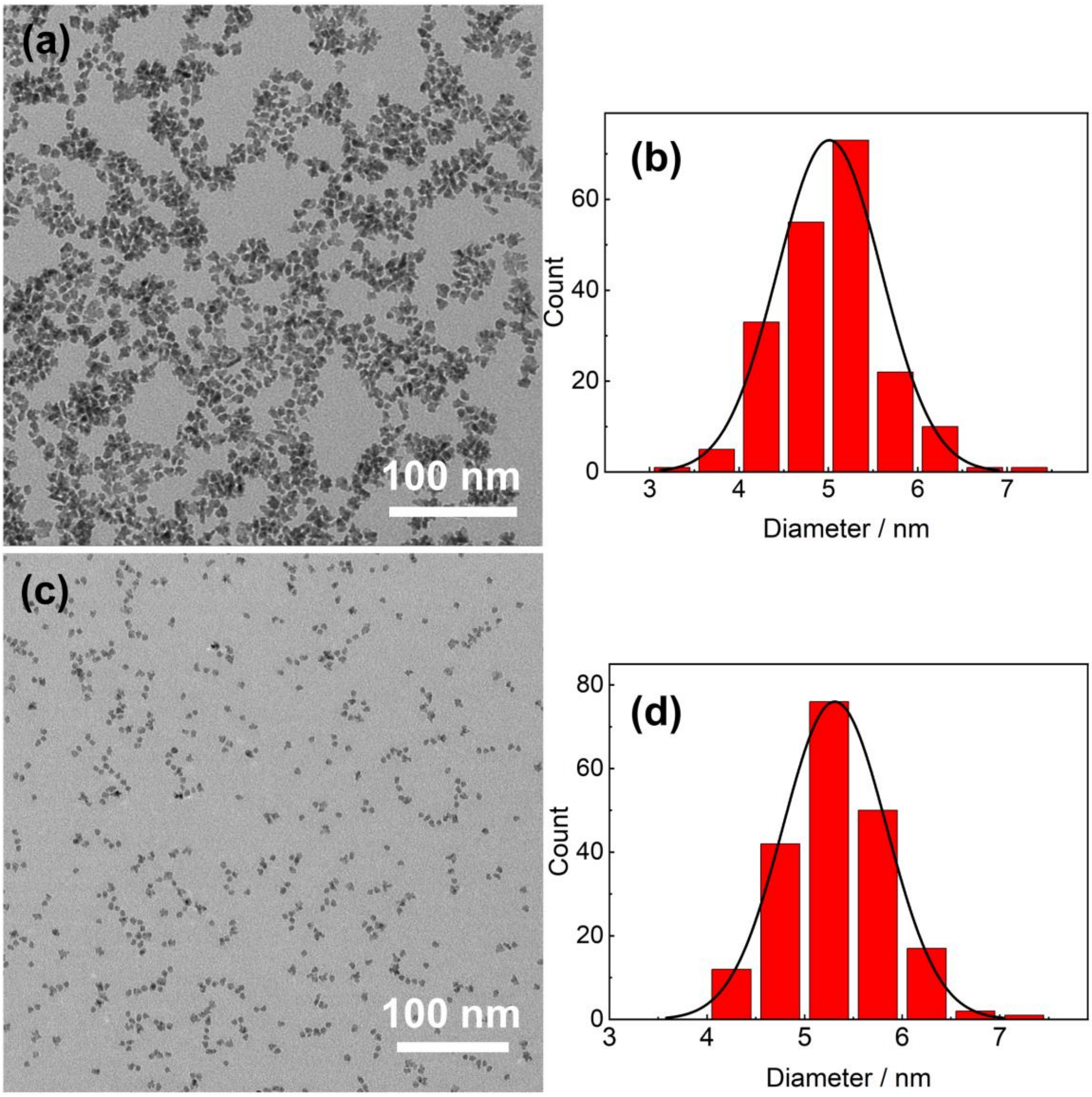

Figure S1. (a) TEM image of $Ru_{0.9}Sn_{0.1}@SnO_x$ NPs. (b) Relative particle size distribution histogram of $Ru_{0.9}Sn_{0.1}@SnO_x$ NPs. (c) TEM image of $Ru_{0.8}Sn_{0.2}@SnO_x$ NPs. (d) Relative particle size distribution histogram of $Ru_{0.8}Sn_{0.2}@SnO_x$ NPs.

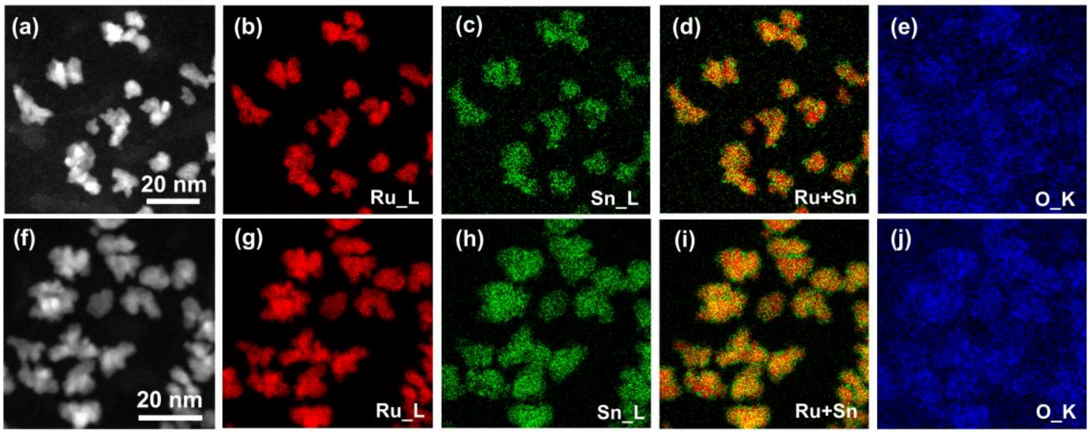

Figure S2. (a) HAADF-STEM image of $Ru_{0.9}Sn_{0.1}@SnO_x$ NPs. Corresponding STEM-EDX maps using the L-line characteristic X-ray of (b) Ru, (c) Sn, (d) overlay of Ru and Sn, and K-Line characteristic X-ray of (e) O. (f) HAADF-STEM image of $Ru_{0.8}Sn_{0.2}@SnO_x$ NPs. Corresponding STEM-EDX maps of (g) Ru, (h) Sn, (i) overlay of Ru and Sn, and (j) O.

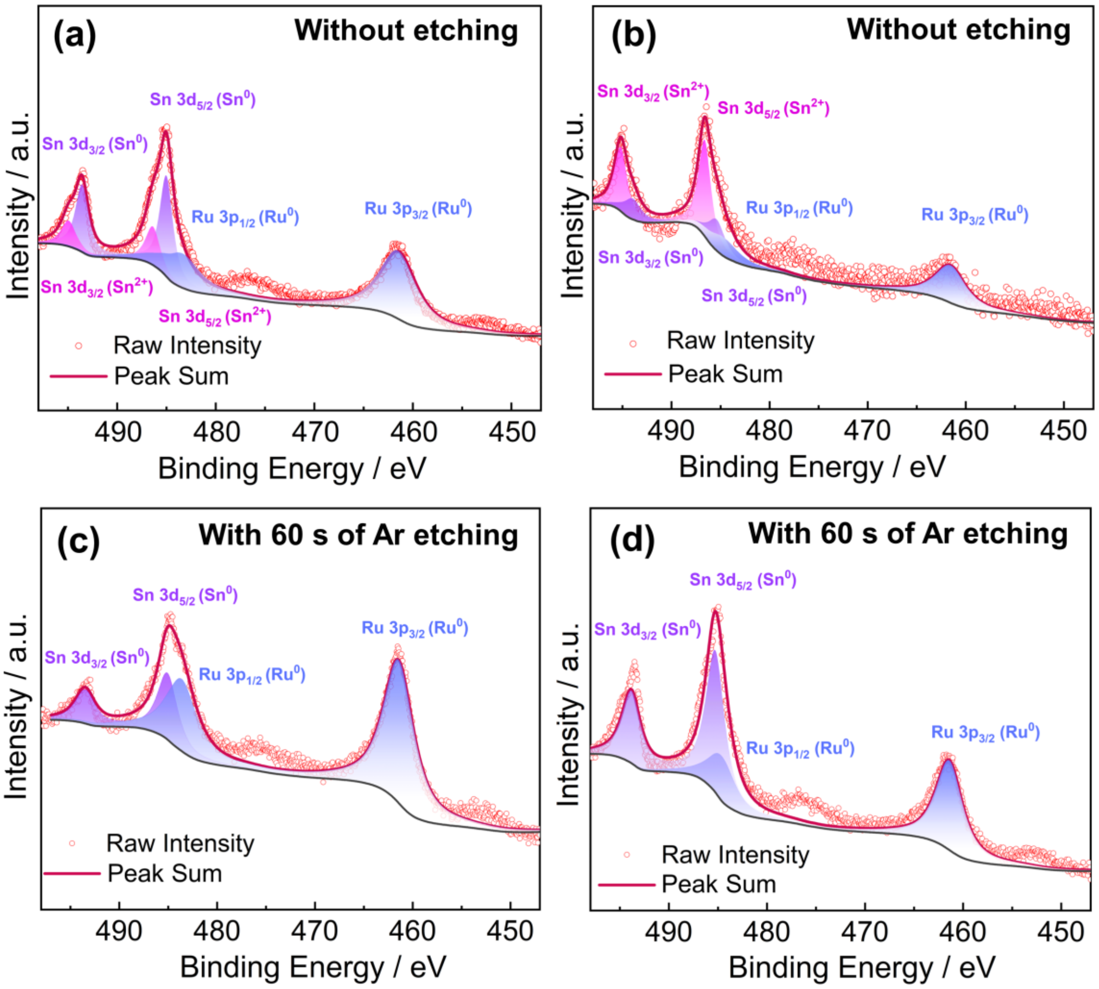

Figure S3. XPS spectra of (a) $Ru_{0.9}Sn_{0.1}@SnO_x$ NPs and (b) $Ru_{0.8}Sn_{0.2}@SnO_x$ NPs without etching. XPS spectra of (c) $Ru_{0.9}Sn_{0.1}@SnO_x$ NPs and (d) $Ru_{0.8}Sn_{0.2}@SnO_x$ NPs with 60 s of Ar etching. The background files are shown as black curves.

Table S1. XPS peak area of Sn $3d_{5/2}$ in $RuSn@SnO_x$ NPs without etching

Sample	Species	Area (%)
$Ru_{0.9}Sn_{0.1}@SnO_x$ NPs	Sn^0	70.54
	Sn^{2+}	29.46
$Ru_{0.8}Sn_{0.2}@SnO_x$ NPs	Sn^0	30.70
	Sn^{2+}	69.30

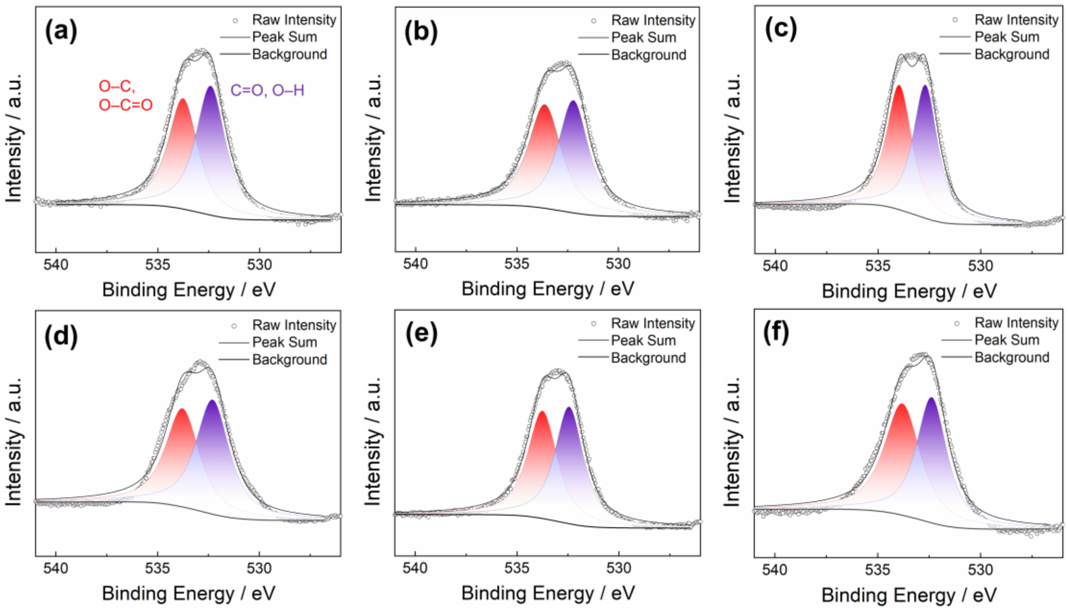

Figure S4. O 1s XPS spectra of (a) $Ru_{0.9}Sn_{0.1}$@SnO_x NPs, (b) $Ru_{0.8}Sn_{0.2}$@SnO_x NPs, (c) carbon black, (d) Ru NPs/C, (e) $Ru_{0.94}Sn_{0.06}$ NPs/C and (f) $Ru_{0.87}Sn_{0.13}$ NPs/C.

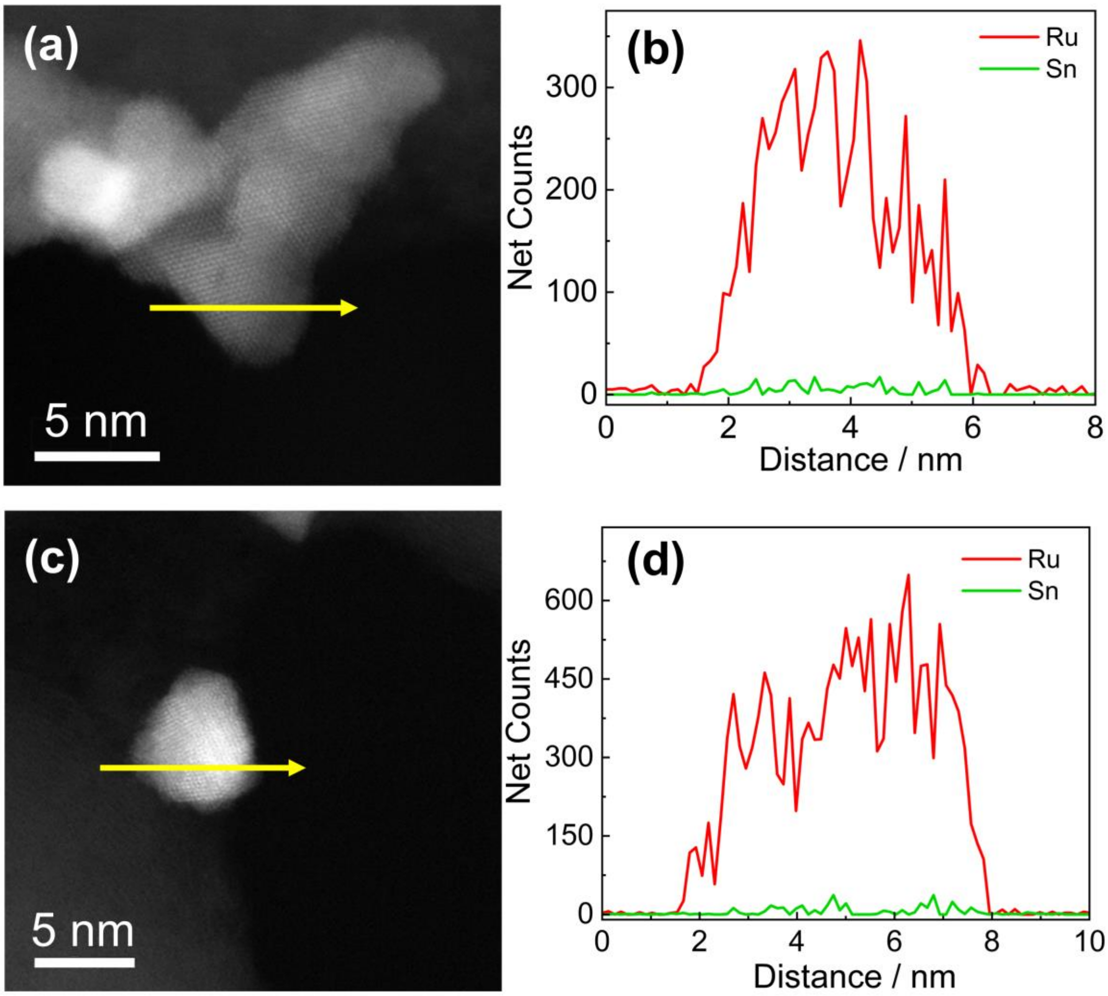

Figure S5. (a) and (c) HAADF-STEM images of $Ru_{0.94}Sn_{0.06}$ NPs/C. (b) and (d) EDX line scan profiles of the $Ru_{0.94}Sn_{0.06}$ NPs/C. The direction of the line scan is indicated by an arrow through the NP.

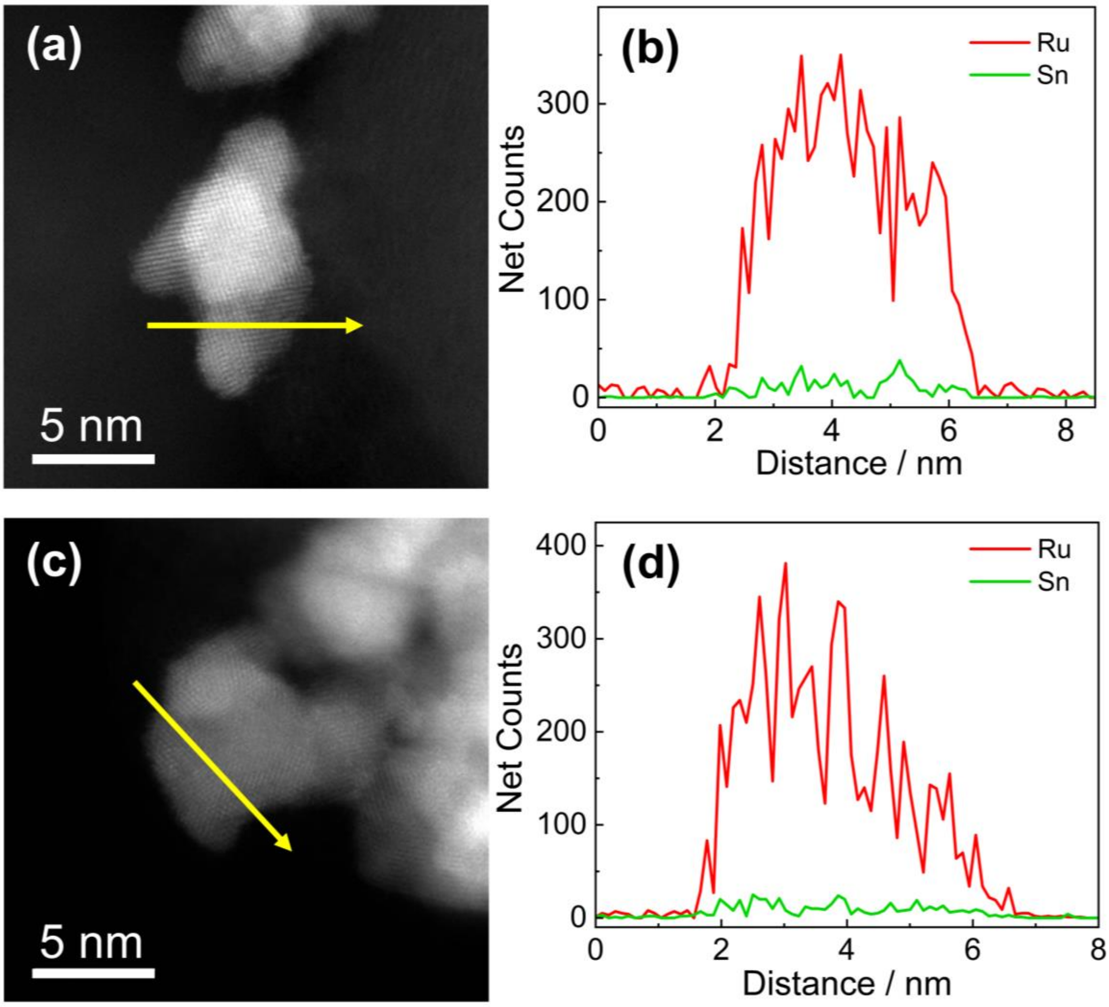

Figure S6. (a) and (c) HAADF-STEM images of $Ru_{0.87}Sn_{0.13}$ NPs/C. (b) and (d) EDX line scan profiles of $Ru_{0.87}Sn_{0.13}$ NPs/C. The direction of the line scan is indicated by an arrow through the NP.

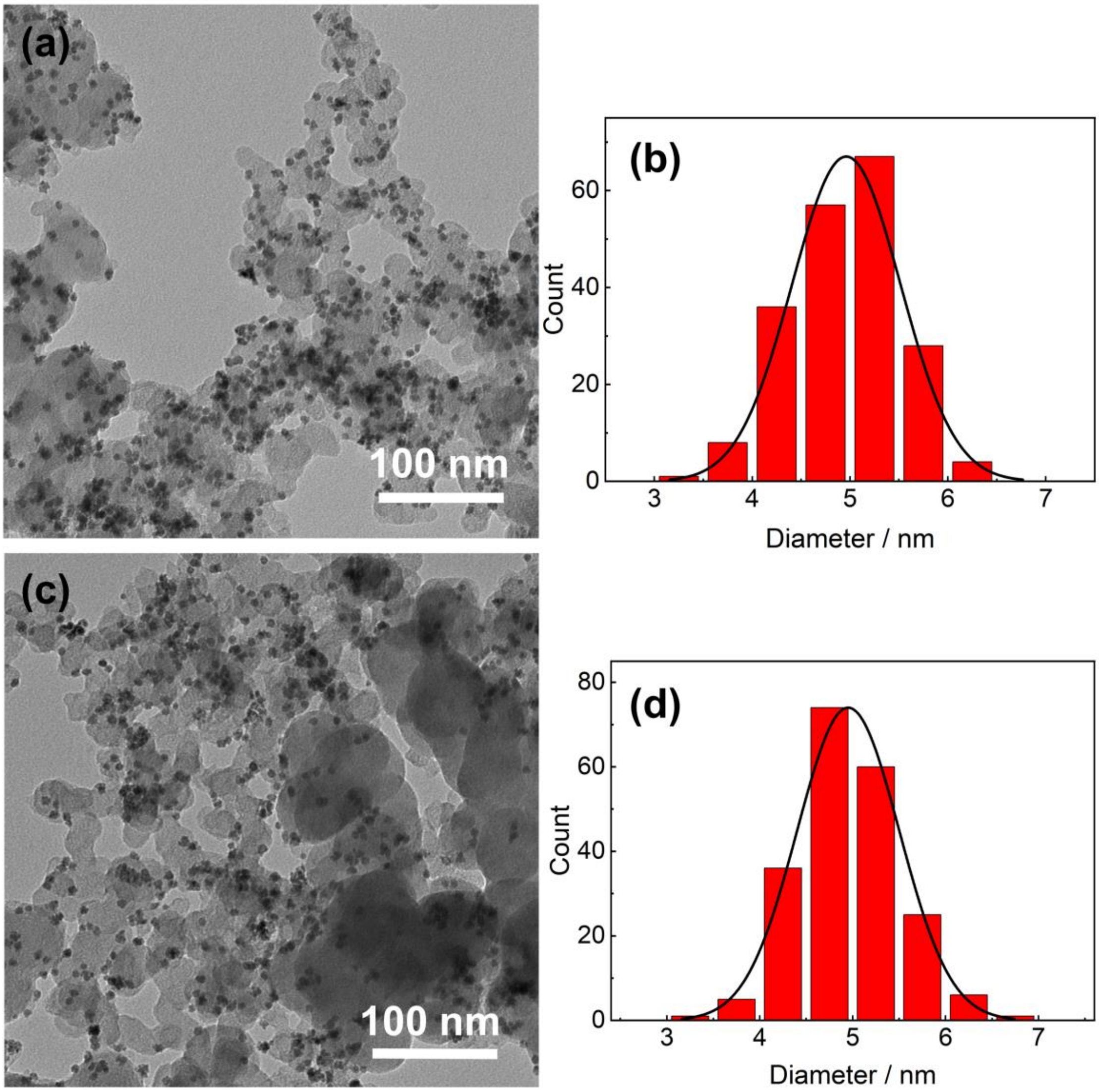

Figure S7. (a) TEM image of $Ru_{0.94}Sn_{0.06}$ NPs/C. (b) Relative particle size distribution histogram of $Ru_{0.94}Sn_{0.06}$ NPs/C. (c) TEM image of $Ru_{0.87}Sn_{0.13}$ NPs/C. (d) Relative particle size distribution histogram of $Ru_{0.87}Sn_{0.13}$ NPs/C.

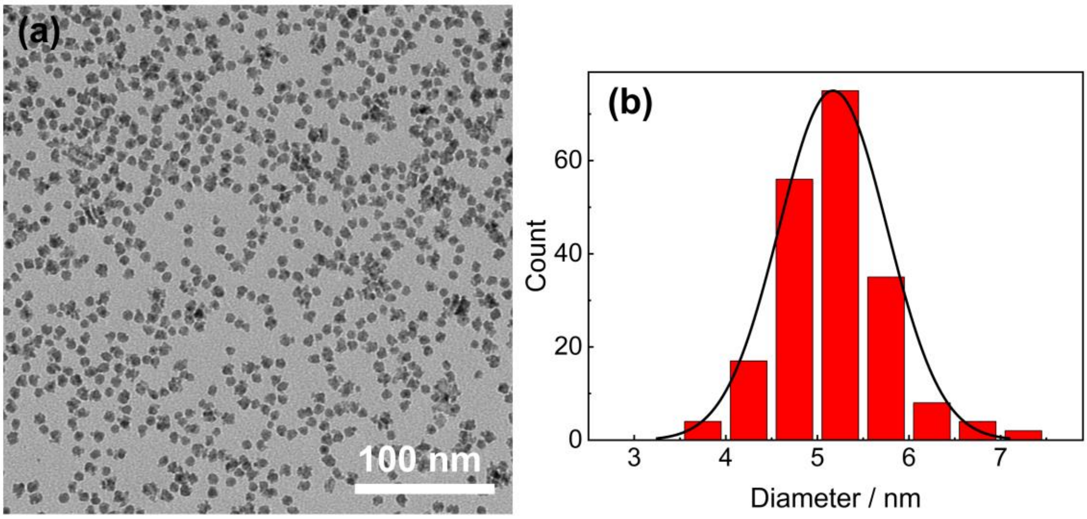

Figure S8. (a) TEM image of Ru NPs. (b) Particle size distribution histogram of Ru NPs.

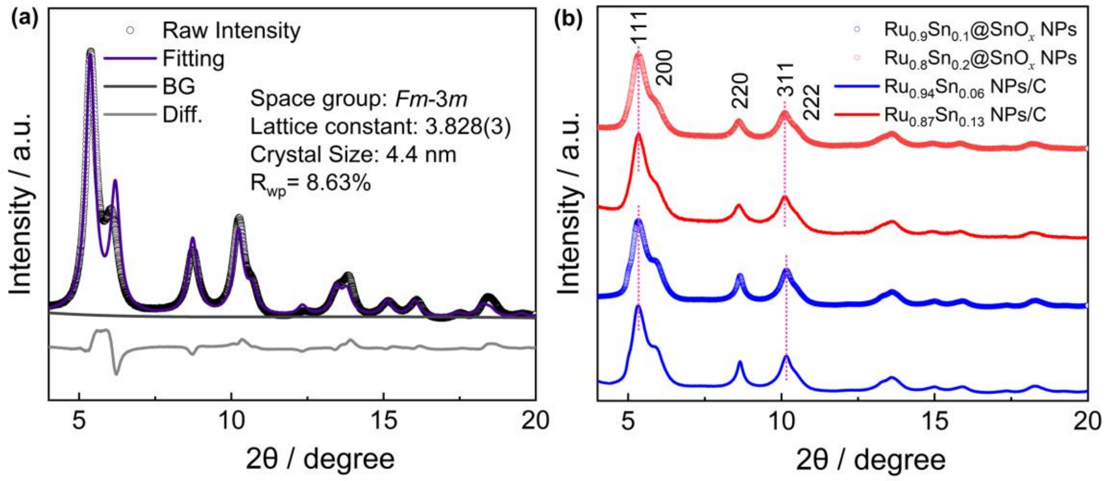

Figure S9. (a) Rietveld refinement analysis of Ru NPs. (b) Synchrotron XRD patterns of RuSn@SnO$_x$ NPs and RuSn NPs/C. The radiation wavelength was 0.2069(1) Å.

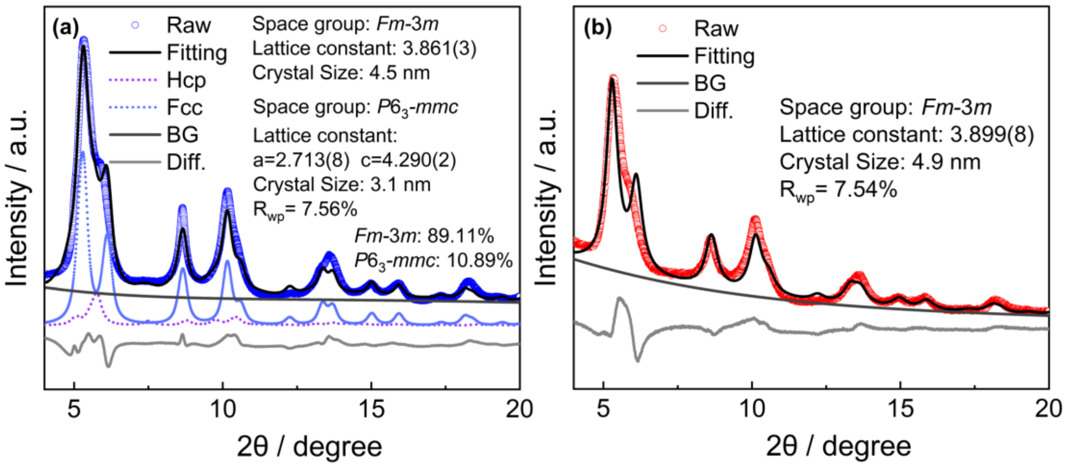

Figure S10. Rietveld refinement analysis of (a) $Ru_{0.94}Sn_{0.06}$ NPs/C and (b) $Ru_{0.87}Sn_{0.13}$ NPs/C.

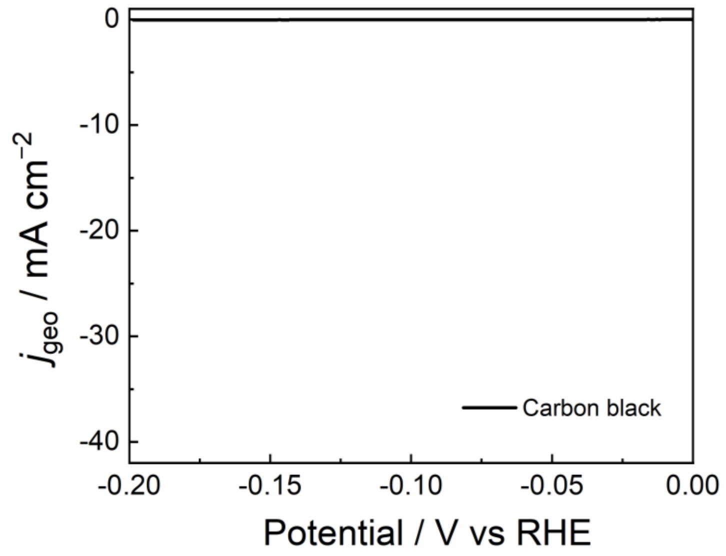

Figure S11. Polarisation curve of HER catalytic performance of carbon black in Ar-saturated 1.0 M KOH.

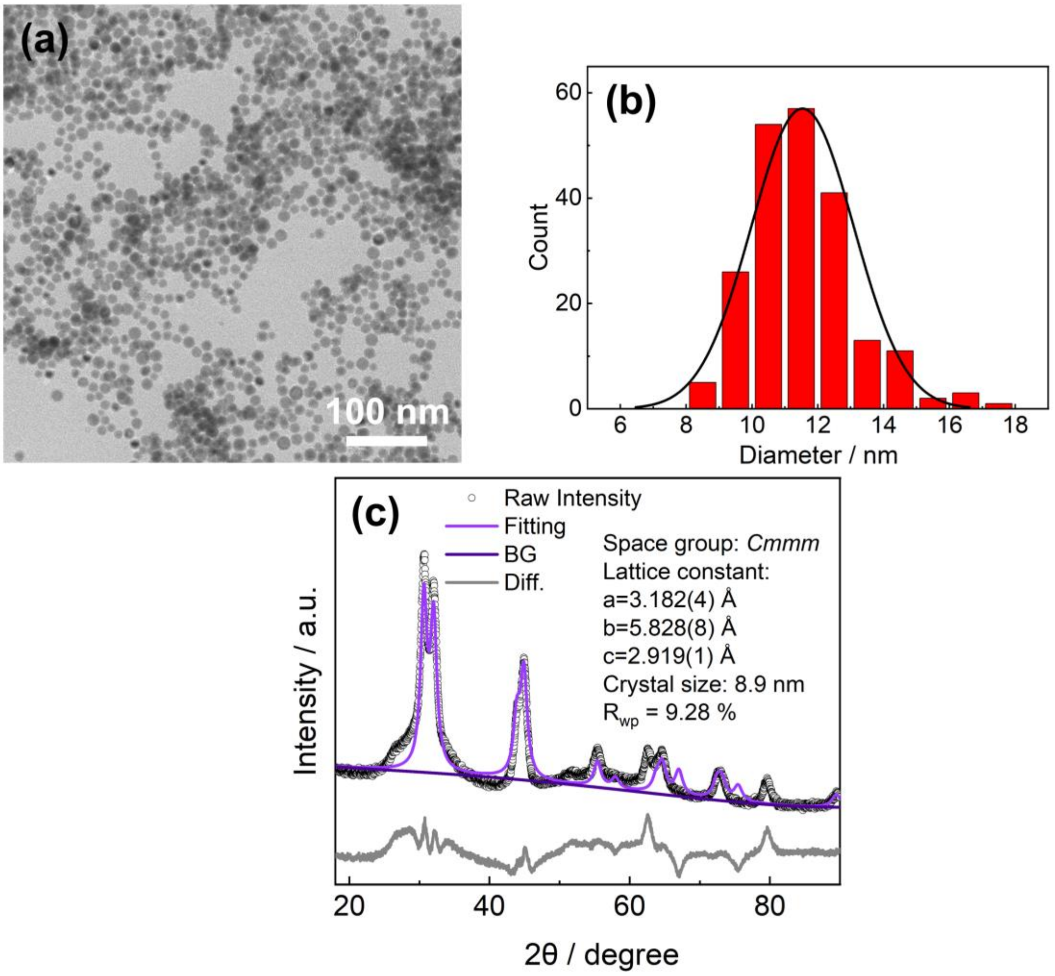

Figure S12. (a) TEM image of Sn NPs. (b) Particle size distribution histogram of Sn NPs. (c) PXRD pattern and Rietveld refinement analysis of Sn NPs. The radiation wavelength was 1.5418(2) Å.

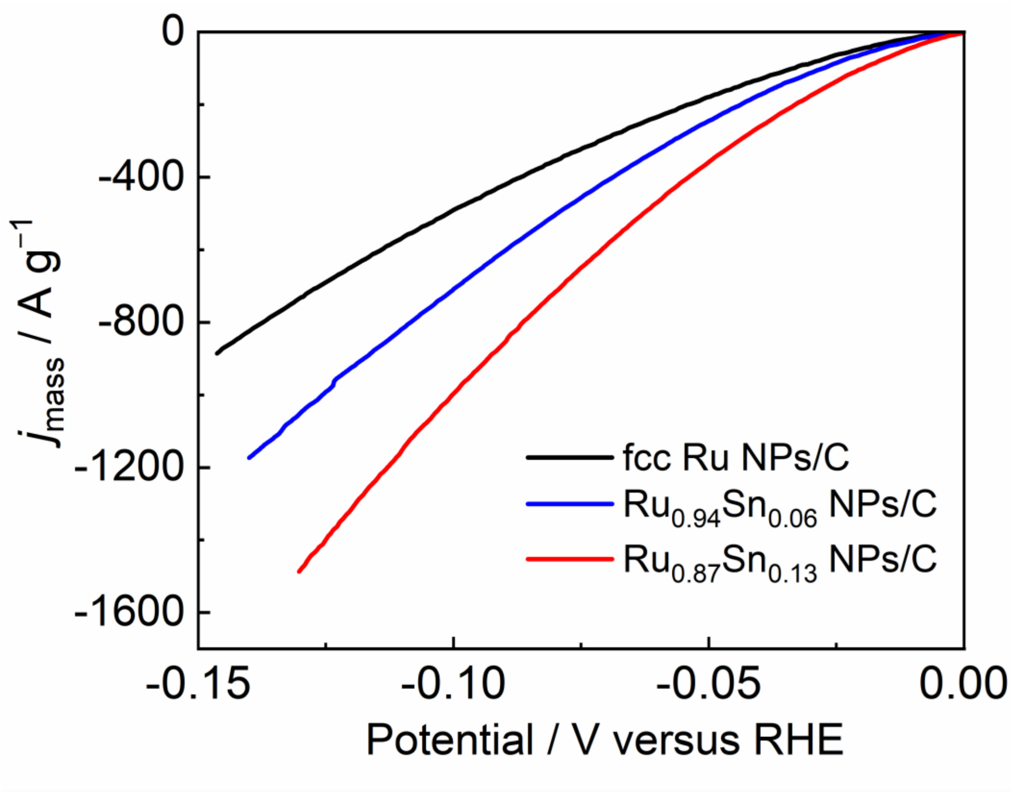

Figure S13. HER mass catalytic activities of Ru NPs/C, $Ru_{0.94}Sn_{0.06}$ NPs/C and $Ru_{0.87}Sn_{0.13}$ NPs/C.

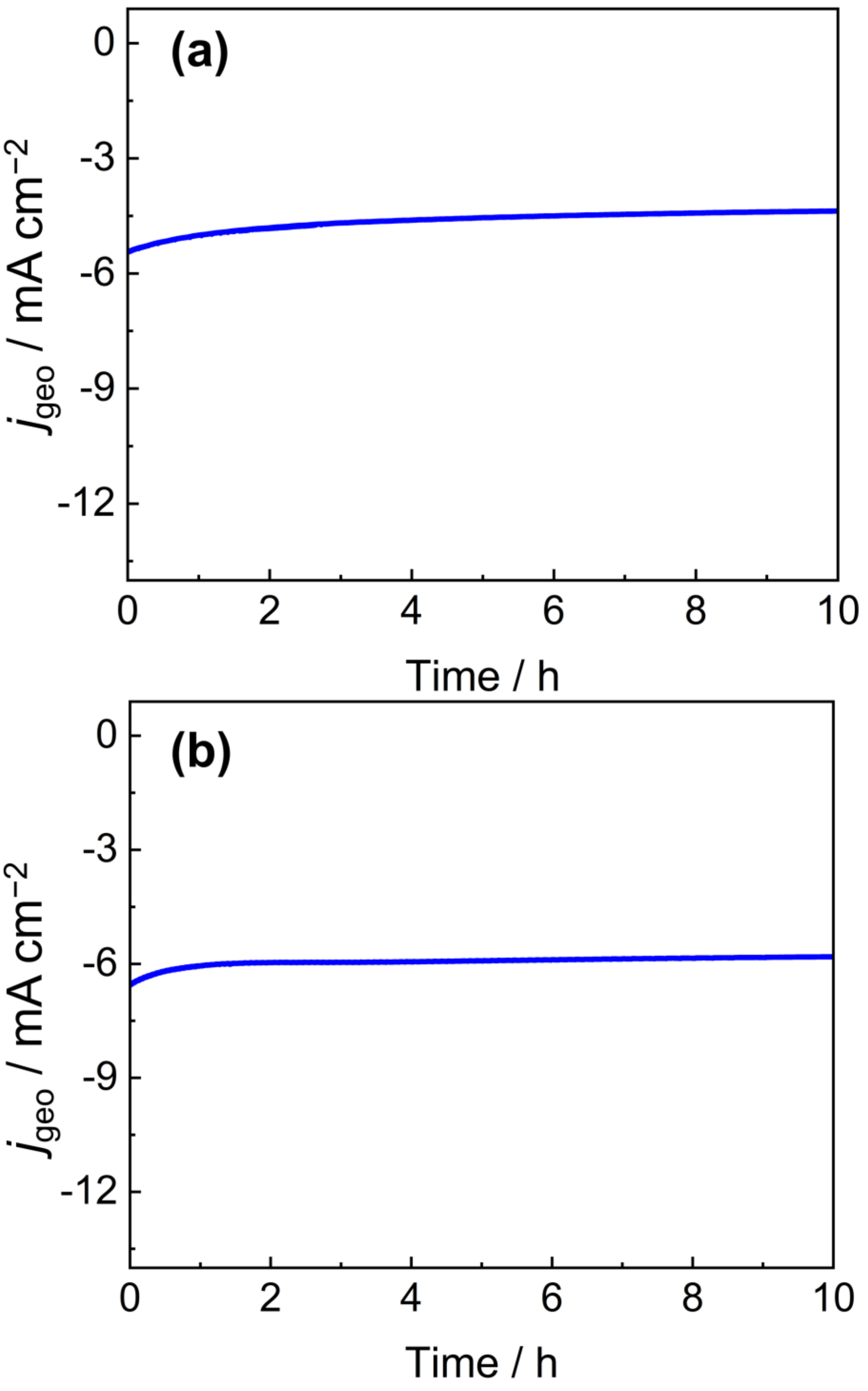

Figure S14. Chronoamperometry curves of (a) $Ru_{0.94}Sn_{0.06}$ NPs/C and (b) $Ru_{0.87}Sn_{0.13}$ NPs/C for HER at −30 mV vs. RHE.

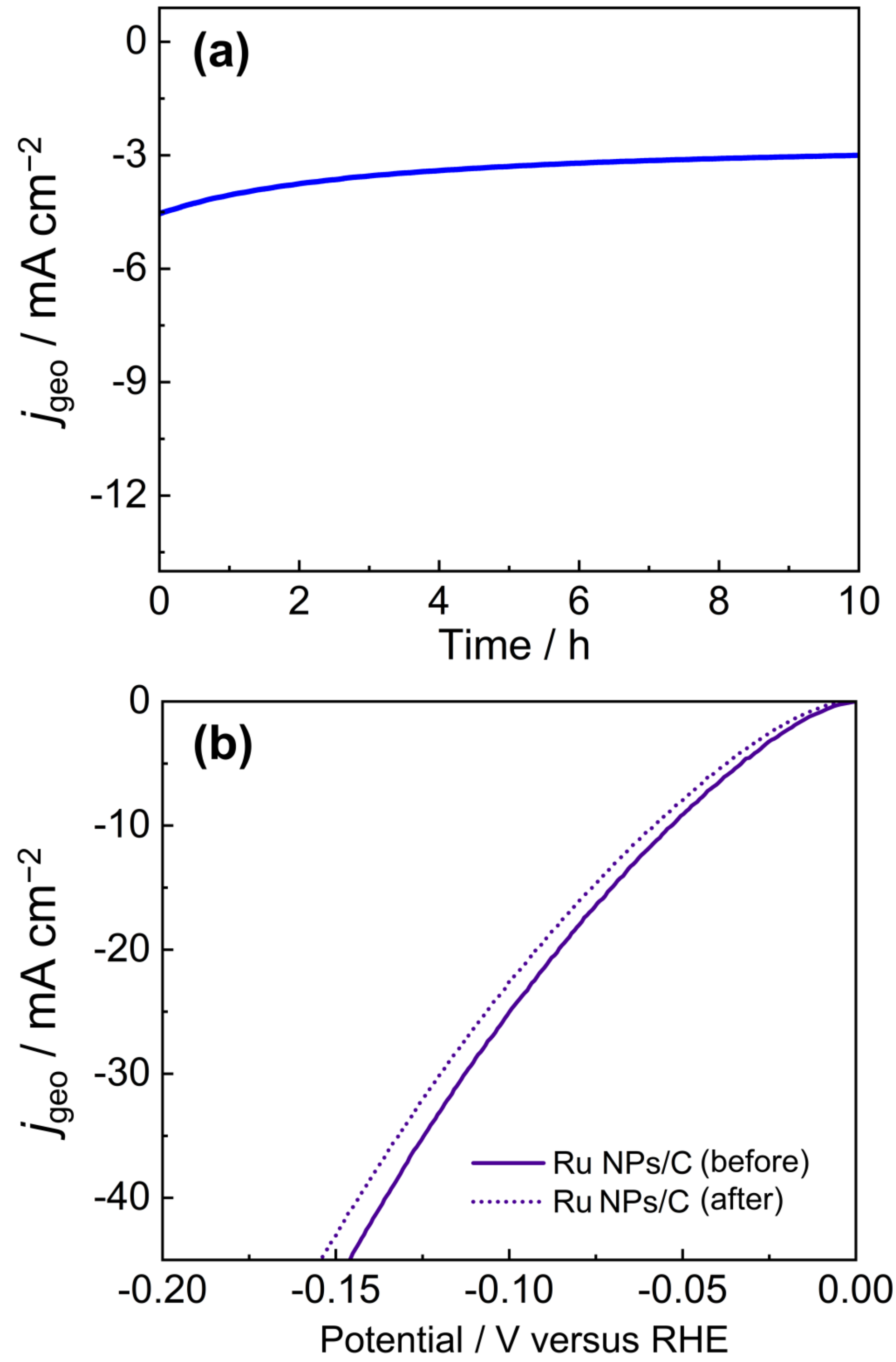

Figure S15. (a) The chronoamperometry curve of Ru NPs/C for HER at −30 mV vs. RHE. (b) Polarisation curves of Ru NPs/C before and after the durability test.

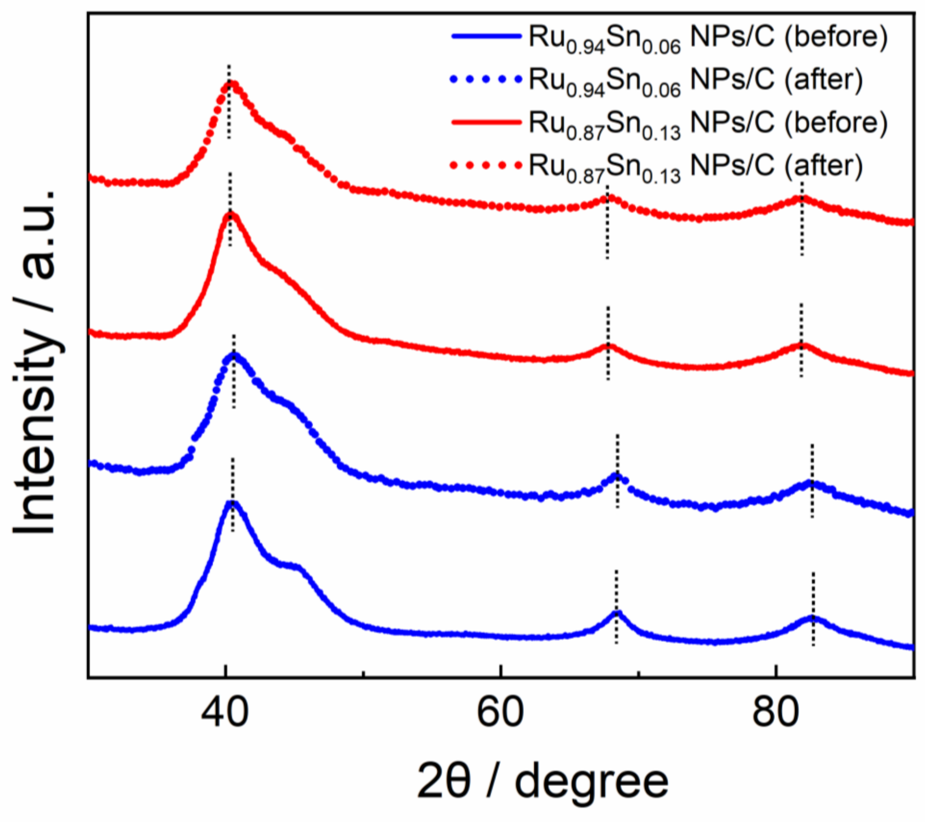

Figure S16. Powder XRD patterns of $Ru_{0.94}Sn_{0.06}$ NPs/C and $Ru_{0.87}Sn_{0.13}$ NPs/C before and after the stability test.

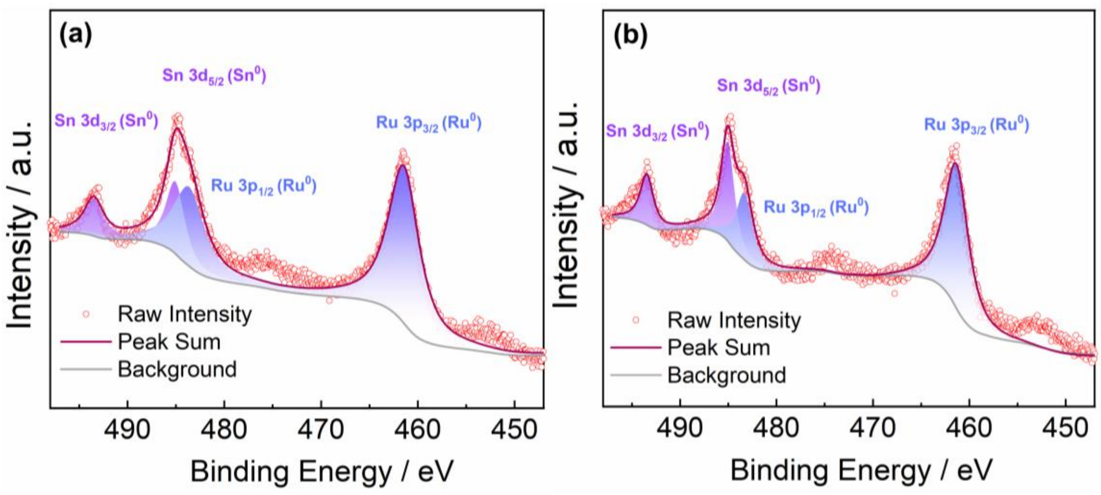

Figure S17. XPS spectra of $Ru_{0.94}Sn_{0.06}$ NPs/C and $Ru_{0.87}Sn_{0.13}$ NPs/C after the stability test.

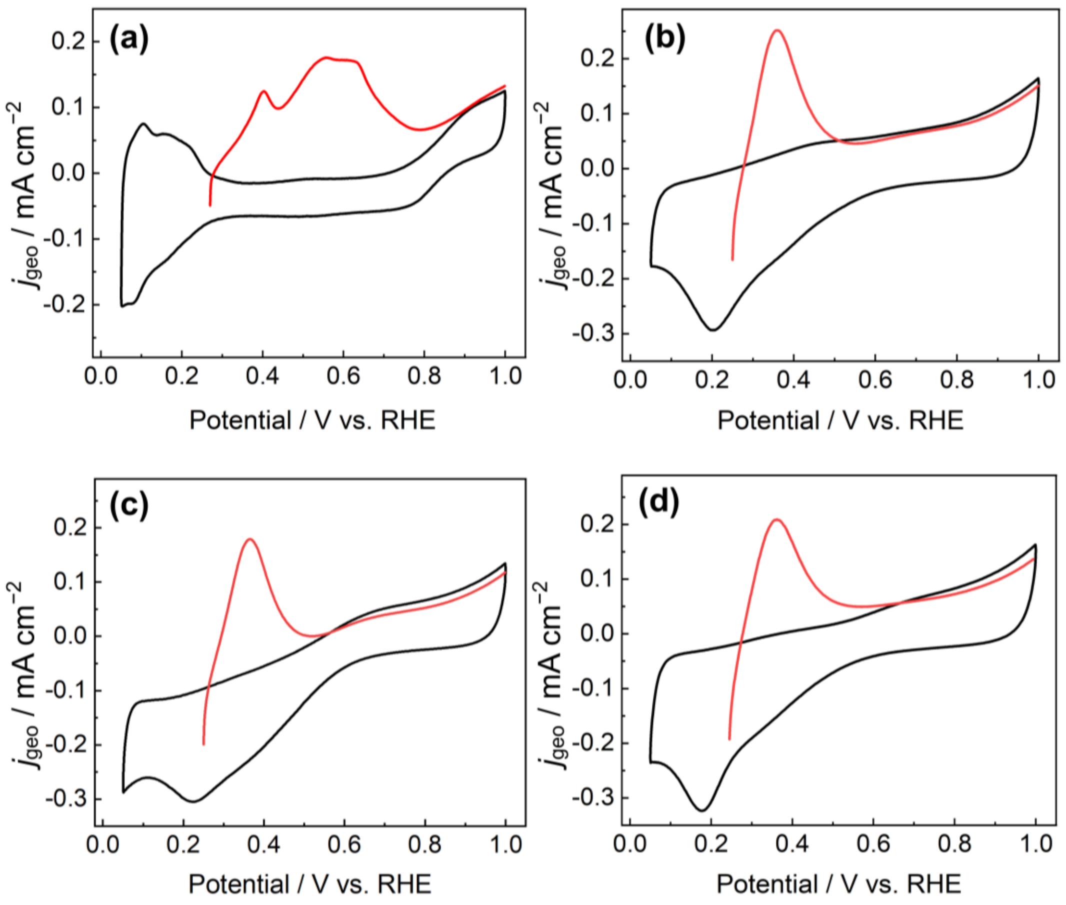

Figure S18. CV curves for ECSA measurement of (a) commercial Pt NPs/C, (b) Ru NPs/C, (c) $Ru_{0.94}Sn_{0.06}$ NPs/C and (d) $Ru_{0.87}Sn_{0.13}$ NPs/C. The black CV curves were obtained in pure Ar-saturated 0.5 M H_2SO_4, and the red CV curves were obtained in Ar-saturated 0.5 M H_2SO_4 with 0.005 M $CuSO_4$.

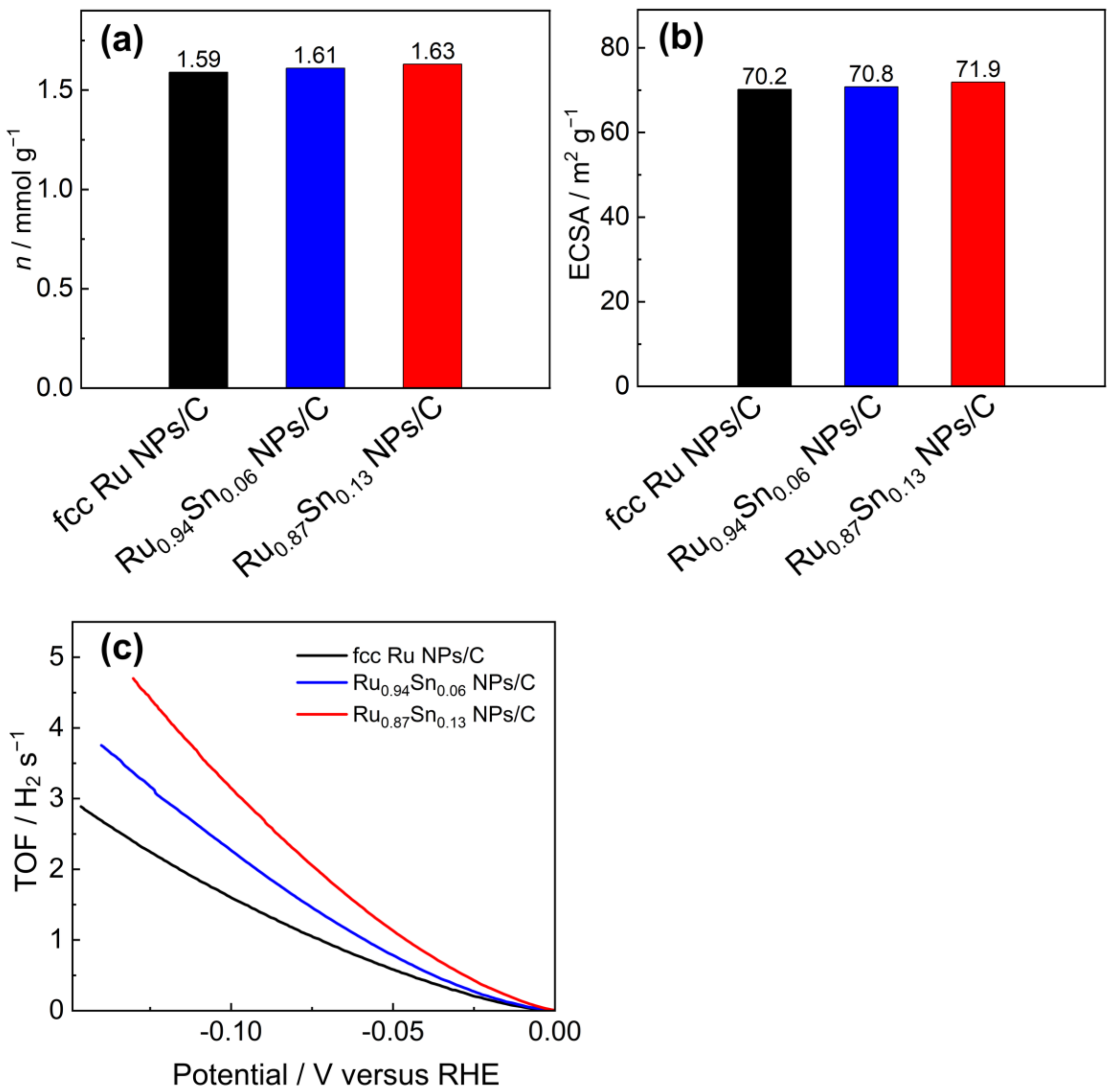

Figure S19. (a) Number of active sites (*n*) of Ru NPs/C, $Ru_{0.94}Sn_{0.06}$ NPs/C and $Ru_{0.87}Sn_{0.13}$ NPs/C. (b) ECSA of Ru NPs/C, $Ru_{0.94}Sn_{0.06}$ NPs/C and $Ru_{0.87}Sn_{0.13}$ NPs/C. (c) TOF values of Ru NPs/C, $Ru_{0.94}Sn_{0.06}$ NPs/C and $Ru_{0.87}Sn_{0.13}$ NPs/C.

Table S2. Comparison of Ru-based catalysts with high HER activity under alkaline conditions

Materials	Electrolyte	Overpotential (10 mA cm^{-2}) /mV	Loading Density/ (mg cm^{-2})	Tafel slope (mV dec^{-1})	References
Ru/Ni_2P NPs	1.0 M KOH	132	0.195	62	10
$Cu_{2-x}S$@Ru NPs	1.0 M KOH	82	0.23	48	11
Sr_2RuO_4	1.0 M KOH	61	0.232	51	12
Ru_2Ni_2 SNs[(a)]	1.0 M KOH	40	0.1	23.4	13
$Ru_{0.33}Se$@TNA[(b)]	1.0 M KOH	57	0.2	50.0	14
RuP_2@NPC[(c)]	1.0 M KOH	52	1.0	38	15
Ru NDs/C[(d)]	1.0 M KOH	43.4	0.177	49	16
Ru–MoS_2/CNT[(e)]	1.0 M KOH	50	1.0	62	17
RuS_x/S-GO[(f)]	1.0 M KOH	58	1.0	56	18
RuNi-NCNFs[(g)]	1.0 M KOH	35	0.612	30	19
Pd_3Ru alloy NPs	1.0 M NaOH	42	0.015	--	20
FeRu alloy NPs	1.0 M KOH	34	0.28	59	21
RuCo@NC[(h)]	1.0 M KOH	34	0.255	36	22
Ru_1@Co/NC[(h)]	1.0 M KOH	103	0.163	210	23
Ru/C_3N_4/C	0.1 M KOH	79	0.204	≈70.8	24
Ru_3Ni_3 NAs[(i)]	1.0 M KOH	39	--	26.9	25
Ru SAs–Ni_2P[(j)]	1.0 M KOH	57	--	75	26
1.38 nm Ru NPs	1.0 M KOH	27	0.007	29	27
subnanometric Ru clusters (Ru-1.0 nm)	1.0 M KOH	13	0.4	25.3	28
20Ru/OMGC[(k)]	1.0 M KOH	30	0.014	30	29
Ru-Mo_2C@CNT[(e)]	1.0 M KOH	15	0.14	26	30
Ru/OMSNNC[(l)]	1.0 M KOH	13	0.4	40.41	31

RuAu-0.2 SAAs[(m)]	1.0 M KOH	24	0.28	37	32
**$Ru_{0.94}Sn_{0.06}$ NPs**	**1.0 M KOH**	**43.41**	**0.128**	**This work**	
**$Ru_{0.87}Sn_{0.13}$ NPs**	**1.0 M KOH**	**33.19**	**0.128**	**This work**	

(a) SNs: sandwiched nanoplates;

(b) TNAs: TiO_2 nanotube arrays;

(c) NPC: N, P dual-doped carbon-encapsulated;

(d) NDs: nanodendrites;

(e) CNT: carbon nanotube;

(f) S-GO: sulfur-doped graphene oxide;

(g) NCNFs: nitrogen-doped carbon nanofibers;

(h) NC: N-doped carbon;

(i) NAs: nanosheet assemblies;

(j) SAs: single-atomic site;

(k) OMGC: ordered mesoporous carbons constructed with graphitic tubular frameworks;

(l) OMSNNC: ordered microporous superstructure of N-doped nanoporous carbon;

(m) SAAs: single-atom alloys.

References

1. X. T. Chen, K. X. Wang, Y. B. Zhai, H. J. Zhang, X. Y. Wu, X. Wei and J. S. Chen, *Dalton Trans.*, 2014, **43**, 3137–3143.
2. L. Deng, F. Hu, M. Ma, S. C. Huang, Y. Xiong, H. Y. Chen, L. Li and S. Peng, *Angew. Chem. Int. Ed.*, 2021, **60**, 22276–22282.
3. M. H. M. T. Assumpção, R. F. B. De Souza, D. C. Rascio, J. C. M. Silva, M. L. Calegaro, I. Gaubeur, T. R. L. C. Paixão, P. Hammer, M. R. V. Lanza and M. C. D. Santos, *Carbon*, 2011, **49**(8), 2842–2851.
4. P. Prieto, V. Nistor, K. Nouneh, M. Oyama, M. Abd-Lefdil and R. Díaz, *Appl. Surf. Sci.*, 2012, **258**, 8807–8813.
5. Y. H. Jo, I. Jung, C. S. Choi, I. Kim and H. M. Lee, *Nanotechnology*, 2011, **22**, 225701.
6. M. A. Quiroz, Y. Meas, E. Lamy-Pitara and J. Barbier, *J. Electroanal. Chem. Interfacial Electrochem.,* 1983, **157**, 165–174.
7. Q. Zhang, K. Kusada, D. Wu, T. Yamamoto, T. Toriyama, S. Matsumura, S. Kawaguchi, Y. Kubota and H. Kitagawa, *J. Am. Chem. Soc.*, 2022, **144**, 4224–4232.
8. Y. Zheng, Y. Jiao, Y. Zhu, L. H. Li, Y. Han, Y. Chen, M. Jaroniec and S. Z. Qiao, *J. Am. Chem. Soc.,* 2016, **138**, 16174–16181.
9. Z. Zhuang, Y. Wang, C. Q. Xu, S. Liu, C. Chen, Q. Peng, Z. Zhuang, H. Xiao, Y. Pan, S. Lu, R. Yu, W. C. Cheong, X. Cao, K. Wu, K. Sun, Y. Wang, D. Wang, J. Li and Y. Li, *Nat. Commun.,* 2019, **10**, 4875.
10. J. Q. Chi, X. Y. Zhang, X. Ma, B. Dong, J. Q. Zhang, B. Y. Guo, M. Yang, L. Wang, Y. M. Chai and C. Liu, *ACS Sustain. Chem. Eng.*, 2019, **7**, 17714–17722.
11. D. Yoon, J. Lee, B. Seo, B. Kim, H. Baik, S. H. Joo and K. Lee, *Small*, 2017, **13**, 1700052.
12. Y. Zhu, H. A. Tahini, Z. Hu, J. Dai, Y. Chen, H. Sun, W. Zhou, M. Liu, S. C. Smith, H. Wang and Z. Shao, *Nat. Commun.*, 2019, **10**, 149.
13. J. Ding, Q. Shao, Y. Feng and X. Huang, *Nano Energy*, 2018, **47**, 1–7.
14. K. Wang, Q. Chen, Y. Hu, W. Wei, S. Wang, Q. Shen and P. Qu, *Small*, 2018, **14**, 1802132.
15. Z. Pu, I. S. Amiinu, Z. Kou, W. Li and S. Mu, *Angew. Chem. Int. Ed.,* 2017, **56**, 11559–11564.
16. K. Gao, Y. Wang, Z. Wang, Z. Zhu, J. Wang, Z. Luo, C. Zhang, X. Huang, H. Zhang and W. Huang, *Chem. Commun.,* 2018, **54**, 4613–4616.
17. X. Zhang, F. Zhou, S. Zhang, Y. Liang and R. Wang, *Adv. Sci.*, 2019, **6**, 1900090.
18. P. Li, X. Duan, S. Wang, L. Zheng, Y. Li, H. Duan, Y. Kuang and X. Sun, *Small*, 2019, **15**, 1904043.
19. M. Li, H. Wang, W. Zhu, W. Li, C. Wang and X. Lu, *Adv. Sci.*, 2020, **7**, 1901833.
20. X. Qin, L. Zhang, G. L. Xu, S. Zhu, Q. Wang, M. Gu, X. Zhang, C. Sun, P. B. Balbuena, K. Amine and M. Shao, *ACS Catal.*, 2019, **9**, 9614–9621.
21. H. J. Kim, E. Hong, Y. Hong, J. Kim, M. K. Kabiraz, Y. M. Kim, H. Lee, W. S. Seo and S. I. Choi, *Nanoscale*, 2023, **15**, 5816–5824.
22. F. Zhang, Y. Zhu, Y. Chen, Y. Lu, Q. Lin, L. Zhang, S. Tao, X. Zhang and H. Wang, *J. Mater. Chem. A*, 2020, **8**, 12810–12820.
23. W. H. Lai, L. F. Zhang, W. B. Hua, S. Indris, Z. C. Yan, Z. Hu, B. Zhang, Y. Liu, L. Wang, M. Liu, R. Liu, Y. X. Wang, J. Z. Wang, Z. Hu, H. K. Liu, S. L. Chou and S. X. Dou, *Angew. Chem. Int. Ed.*, 2019, **58**, 11868–11873.
24. Y. Zheng, Y. Jiao, Y. Zhu, L. H. Li, Y. Han, Y. Chen, M. Jaroniec and S. Z. Qiao, *J. Am. Chem.*

Soc., 2016, **138**, 16174–16181.
25. J. Yang, Q. Shao, B. Huang, M. Sun and X. Huang, *iScience*, 2019, **11**, 492–504.
26. K. Wu, K. Sun, S. Liu, W. C. Cheong, Z. Chen, C. Zhang, Y. Pan, Y. Cheng, Z. Zhuang, X. Wei, Y. Wang, L. Zheng, Q. Zhang, D. Wang, Q. Peng, C. Chen and Y. Li, *Nano Energy*, 2021, **80**, 105467.
27. D. S. Baek, H. Y. Lim, J. Kim, J. Lee, J. S. Lim, D. Kim, J. H. Lee, J. W. Jang, S. K. Kwak and S. H. Joo, *ACS Catal.*, 2023, **13**, 13638–13649.
28. Q. Hu, K. Gao, X. Wang, H. Zheng, J. Cao, L. Mi, Q. Huo, H. Yang, J. Liu and C. He, *Nat. Commun.*, 2022, **13**, 3958.
29. D. S. Baek, K. A. Lee, J. Park, J. H. Kim, J. Lee, J. S. Lim, S. Y. Lee, T. J. Shin, H. Y. Jeong, J. S. Son, S. J. Kang, J. Y. Kim and S. H. Joo, *Angew. Chem. Int. Ed.*, 2021, **133**, 1461–1469.
30. X. Wu, Z. Wang, D. Zhang, Y. Qin, M. Wang, Y. Han, T. Zhan, B. Yang, S. Li, J. Lai and L. Wang, *Nat. Commun.*, 2021, **12**, 4018.
31. Y. L. Wu, X. Li, Y. S. Wei, Z. Fu, W. Wei, X. T. Wu, Q. L. Zhu and Q. Xu, *Adv. Mater.*, 2021, **33**, 2006965.
32. C. H. Chen, D. Wu, Z. Li, R. Zhang, C. G. Kuai, X. R. Zhao, C. K. Dong, S. Z. Qiao, H. Liu and X. W. Du, *Adv. Energy Mater.*, 2019, **9**, 1803913.
